# Discovery of interesting new polymorphisms in a sugar beet (elite $\times$ exotic) progeny by comparison with an elite panel

**DOI:** 10.1007/s00122-019-03406-0

**Published:** 2019-09-04

**Authors:** Prune Pegot-Espagnet, Olivier Guillaume, Bruno Desprez, Brigitte Devaux, Pierre Devaux, Karine Henry, Nicolas Henry, Glenda Willems, Ellen Goudemand, Brigitte Mangin

**Affiliations:** 1 LIPM, Université de Toulouse, INRA, CNRS, Castanet-Tolosan, France; 2 Florimond Desprez Veuve & Fils SAS, BP41, 3, Rue Florimond Desprez, 59242 Capelle-en-Pévèle, France; 3 SESVanderHave, Industriepark Soldatenplein Zone 2/Nr 15, 3300 Tienen, Belgium

**Keywords:** Sugar beet, Exotic accession, Genetic diversity, QTL detection

## Abstract

**Key message:**

The comparison of QTL detection performed on an elite panel and an (elite $\times$ exotic) progeny shows that introducing exotic germplasm into breeding programs can bring new interesting allelic diversity.

**Abstract:**

Selection of stable varieties producing the highest amount of extractable sugar per hectare (ha), resistant to diseases, and respecting environmental criteria is undoubtedly the main target for sugar beet breeding. As sodium, potassium, and $\alpha$-amino nitrogen in sugar beets are the impurities that have the biggest negative impact on white sugar extraction, it is interesting to reduce their concentration in further varieties. However, domestication history and strong selection pressures have affected the genetic diversity needed to achieve this goal. In this study, quantitative trait locus (QTL) detection was performed on two populations, an (elite $\times$ exotic) sugar beet progeny and an elite panel, to find potentially new interesting regions brought by the exotic accession. The three traits linked with impurities content were studied. Some QTLs were detected in both populations, the majority in the elite panel because of most statistical power. Some of the QTLs were colocated and had favorable effect in the progeny since the exotic allele was linked with a decrease in the impurity content. A few number of favorable QTLs were detected in the progeny, only. Consequently, introgressing exotic genetic material into sugar beet breeding programs can allow the incorporation of new interesting alleles.

**Electronic supplementary material:**

The online version of this article (10.1007/s00122-019-03406-0) contains supplementary material, which is available to authorized users.

## Introduction

The sugar beet (*Beta vulgaris* L. ssp. vulgaris) is an important European crop for sugar production; it is also used as a source for bioethanol and animal feed. It is one of the youngest domesticated crops and probably originated from a relatively limited range of fodder beet types approximately 200 years ago (Fischer 1989). The selection of varieties in the first half of the nineteenth century was a mass selection, and sugar beets were grouped together according to their similarities (Desprez and Desprez 2015). Shortly before the 1860s, in addition to the visual and weight aspects, beets were classified according to their sugar content. In 1856, Louis de Vilmorin set up genealogical selection by taking into account the pedigree and value of the offspring. This type of selection is more accurate than mass selection because it is less affected by environmental effects; consequently, this has allowed for great progress, particularly for complex characters such as yield. Many interesting characteristics, such as monogermy, maintenance of cytoplasmic male sterility, or resistance to the beet necrotic yellow vein virus, have been gradually introgressed into cultivated lines, leading to an annual increase of sugar yield of 2% per ha: 15t/ha produced in 2015, whereas only 700 kg/ha was produced in 1802 (Desprez and Desprez 2015). Currently, the sugar demand has increased with agroethanol development and the increase in worldwide sugar consumption. Therefore, the French sugar beet industry has to be more competitive. An investment program for the future, called AKER (2012–2020), aims to double the annual rate of progress through genetic improvement (http://www.aker-betterave.fr/en/). AKER proposes to increase the genetic variability of sugar beets by searching for interesting new alleles from exotic resources around the world. The introgression of these new alleles in elite material will produce new varieties with a high potential for use by the industry. Wild crop relatives are a source of potentially adaptive genetic diversity for crop breeding programs (Tanksley and McCouch 1997; Monteiro et al. 2018). The wild and cultivated relatives of sugar beets are included in *Beta* section *Beta* (Andrello et al. 2016). In the AKER project, a core collection of 16 accessions has been selected from among the 10,000 accessions maintained by public genebanks worldwide and composed of a wide variety of wild accessions, using passport data, geographic origins, pedigrees, and genotyping data. The first step was maximizing geographic diversity by removing duplicates, going from 10,000 to 2000 exotic accessions. Then, the complementary genetic diversity of elite lines was maximized and the number went from 2000 to 16 exotic accessions. These 16 exotic plants have therefore been selected worldwide as representing the maximum of the genetic variability not present in cultivated lines. One of these accessions was crossed several times with an elite to create a population called (elite $\times$ exotic) progeny. The goal of our study was to determine whether new and interesting allele diversity could be found in this (elite $\times$ exotic) progeny compared to an elite panel. Therefore, the sodium, potassium, and $\alpha$-amino nitrogen contents were phenotyped in the progeny and in an elite panel. Indeed, the recovery of crystalline sugar in the factory depends on the composition of the sugar beet root, and sodium, potassium, and $\alpha$-amino nitrogen are the major melassigenic substances. They increase the solubility of sucrose and thereby reduce the crystallization (Hoffmann 2010), such that the sugar beet quality decreases. It is interesting to search alleles associated with a decrease in these impurities to improve white sugar extraction. Quantitative trait locus (QTL) detections were performed using the progeny and an elite panel for these impurity traits. The comparison of QTLs found in both populations and their positive or negative effects on impurities will allow us to determine whether the progeny has interesting alleles not present in the elite panel. Looking for exotic QTLs that improve traits of interest has already been done successfully in other species. For example, Nedelkou et al. (2017) created a tri-parental population in wheat, a progeny from two cultivated lines and one exotic donor accession, and demonstrated that two detected exotic QTLs had a substantial favorable effect on the studied traits. In (Schnaithmann and Pillen 2013), favorable exotic QTLs were found in a barley introgression line. These studies confirm the potential of exotic germplasm to induce interesting traits in cultivated lines of different species.

## Materials and methods

### (elite $\times$ exotic) progeny

#### Plant materials

An exotic accession of *Beta vulgaris maritima* from Denmark was crossed with a sugar beet elite pollinator (*Beta vulgaris* L.) from the Florimond Desprez company. Two successive backcrosses with another elite pollinator also from the same company were then completed, leading to 187 individuals that constituted the (elite $\times$ exotic) progeny.

#### Phenotypic data

The (elite $\times$ exotic) progeny was evaluated in 2016. A total of 187 individuals were evaluated in combination with a tester MSF1 and compared to four commercial hybrids and elite accessions as checks. The entire progeny was evaluated in a lattice design with two replicates for productivity and impurity traits in nine locations: AVE607, BAR601, BEL601, BER601, DOM601, MEM601, and PIE601 in France, DAW601 in Great Britain, and UPI601 in Belgium. The three measured impurity traits were the sodium content (Na, meq/100 g) and potassium content (K, meq/100 g) measured by a flame photometer, and the $\alpha$-amino nitrogen content (N, meq/100 g) measured by colorimetry. The measured traits linked with productivity were the root yield (RY, tons/ha) and the sucrose content (S, %) measured by refractometry. Other traits linked with productivity were calculated according to the impurities: the white sugar (WS) as $\text {WS} = S-(0.14 * ((\text {K} + \text {Na}) + 0.25 * \text {N} + 0.5)$, and the white sugar yield (WSY) as $\text {WSY} = ((\text {RY} * \text {WS}) / 100$). Data linked with the productivity are not publicly available at this moment.

#### Phenotypic data analysis

Spatial effects on each of the nine progeny environments were adjusted with the R package SpATS Rodríguez-Álvarez et al. (2017), available from CRAN (https://CRAN.R-project.org/package=SpATS). The SpATS method allows us to consider the local trends, thanks to a smooth bivariate function *f*(*u*, *v*) represented by 2D P splines, where *u* is the numeric vector of rows and *v* is the numeric vector of columns. For this experiment, additional terms were included in the SpATS model to account for other sources of environmental variation and genotype effects. We assumed a model including random independent factors for rows ($\varvec{c}_r$), columns ($\varvec{c}_c$), and genotypes ($\varvec{c}_g$), and also fixed factors for genetic checks ($\varvec{\beta }_t$), repetitions ($\varvec{\beta }_n$), and the spatially independent error term $\varvec{e} \sim \mathcal {N}(0,\sigma _e^2\varvec{I})$. Adapting the formulation in Rodríguez-Álvarez et al. (2016) in order to analyze a single trial and to include repetitions, the SpATS mixed model for each trial is:

$$\begin{aligned} y = f(\varvec{u}, \varvec{v}) + \varvec{Z}_r\varvec{c}_r + \varvec{Z}_c\varvec{c}_c + \varvec{Z}_g\varvec{c}_g + \varvec{X}_t\varvec{\beta }_t + \varvec{X}_n\varvec{\beta }_n + \varvec{\epsilon } \end{aligned}$$

where *y* is the adjusted phenotype vector, and $\varvec{Z}_r$, $\varvec{Z}_c$, $\varvec{Z}_g$, $\varvec{X}_t$ and $\varvec{X}_n$ are the design matrices associated with rows, columns, genotypes, genetic checks, and repetitions, respectively.

The generalized heritability was also computed with the SpATS package.

Before computing the mean phenotype, we looked at the distribution of adjusted traits in each environment (see boxplots in supplementary material, Figs. S1–S3). We can notice that the potassium content was low in BAR601 and MEM601, the sodium content was particularly high in UPI601, and the $\alpha$-amino nitrogen content was high in MEM601 and particularly high in UPI601. UPI601 was in Belgium, where nitrogen needs were greater than that in France and Great Britain. The role of nitrogen is of high importance as it affects N and Na concentrations in sugar beet roots (Tsialtas and Maslaris 2005). Phenotype values were therefore impacted by the technical itinerary in this environment. A principal component analysis (PCA) of the nine environments according to all the traits evaluated in the progeny (productivity, impurities) shows that the three above environments were far from the six others (see in supplementary material Fig. S4). We wanted to detect QTLs on the mean phenotype representative of a mean stressed environment. UPI601, which had a particular itinerary, and BAR601 and MEM601 which seem extreme were therefore removed from the study. Only the six consistent environments were kept for further analyses, and the mean phenotype for each trait was then calculated as the mean of the trait value in these six adjusted environments. This mean phenotype was used to find generalist SNPs.

#### Genotyping

A proprietary 35K Axiom^®^ beet genotyping array was developed along with Affymetrix (http://www.affymetrix.com), CA (USA). This array carried 33,621 high-quality SNPs from which 88% were recently generated through next-generation sequencing of the 16 beet accessions, selected into the AKER project. The SNPs put on the chip were chosen in respect to the technical constraints required by Affymetrix and to be distributed homogeneously along the genome. In brief, the NGS reads ($2 \times 100$ bp paired-end) were mapped onto the sugar beet reference genome (Dohm et al. 2014). SNP calling was performed using a classical pipeline of Burrows–Wheeler Aligner (BWA), samtools, mpileup, varscan, and perl scripts. For genotyping, approximately 30 mg of fresh leaf tissue of each individual was sampled in a 96-deep well, immediately placed at $-80\,^{\circ }\hbox {C}$ for at least 24 h and then lyophilized for 48 h. The freeze-dried leaves were subsequently ground using a MM400 Retsch grinder (http://www.retsch.com) for 150 s at 30 frequencies per s Magnetic bead DNA extraction was performed on a robotized platform. All individuals were genotyped using the GeneTitan®microarray automated scanner following the manufacturer’s recommendations (http://www.affymetrix.com/support/technical/byproduct.affx?product=genetitan). Genotyping crude data were analyzed with the Axiom^®^ 1.1. analysis suite software package (http://www.affymetrix.com/support/technical/byproduct.affx?product=axiomanalysissuite). For further analyses, only the highest quality SNPs corresponding to “Poly High Resolution” and “No Minor Homozygote” categories were used.

Only SNPs whose parental allele provenance was known without ambiguity were kept. The missing genomic data were then imputed for each linkage group using Beagle software (Browning and Browning 2009), leading to 1638 SNPs. After this imputation, some SNPs had exactly the same genetic information. The redundancy of information was not useful for further GWAS analyses; on the contrary, it increased the computational burden and could skew the calculation of the relatedness between hybrids, decreasing the power in regions with many redundant markers (Rincent 2014). Thus, redundant SNPs were discarded. Then, a minor allele frequency (MAF) filter was used to remove SNPs with MAF less than 0.03. Finally, only SNPs with three genotypic classes were retained, coded as 0, 1, or 2 for homozygous for the elite allele, heterozygous, or homozygous for the exotic allele, respectively. All the above filtration steps lead to 604 SNPs retained for subsequent analyses.

### Elite panel

#### Plant materials

A panel of 2101 elite lines of diploid sugar beets (*Beta vulgaris* L.), resulting from many different crosses in Florimond Desprez’s breeding program, was analyzed in this study. This population was already studied in Mangin et al. (2019).

#### Phenotypic data

This panel was evaluated in testcrosses in company multi-environment trials (MET) in 2009, 2010, and 2011 as described in supplementary material of Mangin et al. (2019). Testcross progenies were produced by crossing each elite line to the same single-cross hybrid as a tester. The evaluated traits were the same for the (elite $\times$ exotic) progeny: the sodium content (Na, meq/100 g) measured by a flame photometer, the potassium content (K, meq/100 g) measured by a flame photometer, and the $\alpha$-amino nitrogen content (N, meq/100 g) measured by colorimetry for impurity traits, and others traits linked with productivity.

#### Phenotypic data analysis

Phenotypes were first adjusted per environment, and then the mean phenotype was calculated as described in supplementary material of Mangin et al. (2019). These mean phenotypes were used as the observed phenotypes for further analyses.

The structure of the elite panel was also analyzed. The optimal cluster number of the hierarchical clustering on principle components analysis was set to two. Clusters contained 676 (Panel A) and 1425 individuals (Panel B).

Boxplots of potassium content, $\alpha$-amino nitrogen content and sodium content in each panel and in the entire population were plotted in supplementary material (see in supplementary material Figs. S5 to S7). Phenotypic variabilities were similar in both panels and in the entire population.

#### Genotyping

The genotyping of this population was completed as described in Mangin et al. (2019). A 0.05 MAF filter was applied, and only SNPs with three genotypic classes were retained, coded 0, 1, and 2 for homozygous for one allele, heterozygous, or homozygous for the other allele, respectively. A total of 626 non-redundant and polymorphic SNPs were retained in the Panel B cluster, and a total of 619 SNPs were retained in the Panel A cluster.

### Genetic map

In the AKER project (http://www.aker-betterave.fr/en/), 16 exotic accessions have been identified as representing the maximum of the genetic diversity not already present in elite lines. Four of these exotic accessions were crossed with an elite line, and two successive backcrosses were realized with the same elite line, leading to four progenies. Each of these progenies was genotyped with the same 33K Axiom^®^ beet genotyping array as the studied (elite $\times$ exotic) progeny.

#### Data cleaning

The 33,621 genotyped SNPs were filtered to remove erroneous data. This filter was applied separately for each population, by the following criteria. First, the SNPs were categorized based on their genotyping quality using the “Ps_Classification” function of the Affymetrix^®^’s SNPolisher R package. We discarded the SNPs that were not in the category “PolyHighResolution” for F1S1 populations or either “PolyHighResolution” or “NoMinor” for BC1 populations. Second, markers with more than 5% missing genotypes or with missing or heterozygous elite genotypes were discarded. Third, the SNPs showing segregation distortion were removed. They were detected using a chi-square test with Mendelian segregation as the null hypothesis and a p value threshold of 0.05. Fourth, the SNPs for which wild and elite alleles were inverted were discarded. These SNPs were detected using the “checkAlleles” function of the R package qtl (Broman et al. 2003) with default parameters. This was necessary because it could result in the creation of two linkage groups per chromosome. We could have inverted these SNPs back instead of discarding them, but we did not judge it necessary because there were few: from 0 to 5 for each population.

#### Genetic map building

Linkage groups were created by transitively grouping markers if the estimated recombination frequency between them was less than 0.35 and if the LOD score was greater than 6. Then, the SNPs that were not grouped and small linkage groups of less than five markers were discarded. This lead to 8 or 9 linkage groups for each population. Each linkage group was attributed to the chromosome on which was located most of its SNPs in a preexisting physic map (Dohm et al. 2014) using a different but overlapping set of markers. For any given population, no chromosome was attributed to more than one linkage group. Linkage groups were very consistent between populations. Only 12 markers among those that were included in a linkage group in at least two different populations were not placed on the same chromosome. These 12 SNPs were removed. Chromosome maps were built using the CarthaGène software (De Givry et al. 2005). The datasets of the four populations were merged using the “dsmergor” command. The redundant markers were merged with “mrkdouble” and “mrkmerges.” Then, the maps were built using the command line “buildfw 0 0 1.” See CarthaGène documentation for more information. This produced a map per chromosome and per population. Marker order was common across populations but distances were distinct. Consensus distances were calculated to simplify the use of the maps. For each map, absent markers were projected on the map. Then, the distances between markers were averaged across the four populations to produce the consensus distances. Three aberrant individuals appeared to have tens of recombinations per chromosome on the maps. We assumed that these individuals did not belong to the populations to which they were assigned. These individuals were discarded, and the maps were rebuilt afterward. Although the distance between subsequent SNPs rarely exceeded a few cM in the produced maps, we unusually observed large distances between SNPs on one extremity of chromosome 3; indeed 6 SNPs spanned across an interval of 65 cM. We assumed that this was an artifact of the map-building algorithm that could not determine a correct position for some markers. Therefore, this part of the map was manually removed. Another internal map propriety of the Florimond Desprez company allowed for the positioning of 91 more SNPs on the consensus map. Tables 3 and 4 present a summary of the produced maps. The density of the consensus map and the density of the genotyped markers in each population are represented in the supplementary material (see in supplementary material Figs. S8a and S8b, respectively).

### QTL detection

Association mapping was performed for the QTL detection in both populations. In the (elite $\times$ exotic) progeny, QTL detection could have been performed using linkage analysis, which allows for the inference of QTL genotypes using all the informative markers. We preferred to use an association mapping method instead of linkage analysis, neglecting the intervals between markers, because we had a dense map with few missing values at each marker both methods give comparable power Rebai et al. (1995). Moreover, this choice allowed us to perform QTL detection using the same method in the two populations: the (elite $\times$ exotic) progeny and the elite panel. Each of the three traits was studied using the adjusted phenotype of the six environments and the mean phenotype for the progeny, and only the mean phenotype for the elite panel. A multi-locus approach with forward selection of SNPs (Segura et al. 2012) was used. At each step of the forward method, a mixed model as proposed by Yu et al. (2006) was evaluated. The variance components of polygenic effects and the residuals were estimated once and a Wald test at each SNP was calculated. The SNP with the smallest p-value was included in the model as a fixed regressor for the next step. The variance attributed to the random polygenic terms decreased when fixed regressors were added to the model; therefore, the forward selection stops when the remaining variances were close to zero. Two models were used: an additive mixed model as proposed by Yu et al. (2006), and an additive and dominance mixed model as proposed by Bonnafous et al. (2018). For the entire elite panel, the two panel clusters were modeled in the structure fixed term but for progeny and for the GWAS within each cluster no structure was added in the model. Genome-wide association studies (GWAS) were conducted using the R package mlmm.gwas available from CRAN (https://CRAN.R-project.org/package=mlmm.gwas).

#### The additive model

The additive model, proposed by Yu et al. (2006), can be written as below: Let $y_i$ denote the adjusted phenotype of the individual *i*. Then, the additive model is

$$\begin{aligned} y_i = \mu + x_i^l \theta _{a}^l + u_i + e_i \quad \text { (A model)} \end{aligned}$$

$x_i^l$ is the centered genotype of the *i*th individual at the *l*th marker locus. In the (elite $\times$ exotic) progeny, the genotype is coded 0, 1, or 2 for homozygous for the elite allele, heterozygous, or homozygous for the exotic allele, respectively. In the elite panel, the genotype is coded 0, 1, or 2 for homozygous for one allele, heterozygous, or homozygous for the other allele, respectively. $\theta _{a}^l$ is the additive effect of the *l*th locus; $u_i$ denotes the random additive polygenic effect; and $e_i$ is the residual error. Let $\varvec{u}$ and $\varvec{e}$ be vectors ($u_i$, $i=1,\ldots ,n$) and ($e_i$, $i=1,\ldots ,n$), respectively. Then $\varvec{u} \sim \mathcal {N}(0,\sigma _u^2\varvec{K_a})$, $\varvec{e} \sim \mathcal {N}(0,\sigma ^2_e\varvec{Id})$, where $\varvec{K_a}$ is a matrix of relative kinship coefficients that define the degree of genetic covariance between a pair of individuals, and $\sigma _u^2$ and $\sigma ^2_e$ are polygenic and residual variances, respectively. The relationship matrix is equivalent to the unscaled kinship matrix described by VanRaden (2008):

$$\begin{aligned} \varvec{K_a}=\varvec{XX'} \end{aligned}$$

where $$\varvec{X}=\left[ x_i^l\right] _{\begin{array}{c} l=1,\ldots ,L \\ i=1,\ldots ,n \end{array}}$$ is the centered matrix of the genotypes.

For the elite panel the structure of the population is also considered, so the model used is:

$$\begin{aligned} y_i = \mu + c_i + x_i^l \theta _{a}^l + u_i + e_i \quad \text { (A model with structure)} \end{aligned}$$

where $c_i$ is the cluster to which the *i*th hybrid belongs.

#### The additive and dominance model

A model including additive and dominance effects of SNPs as proposed in Bonnafous et al. (2018) was also used. The additive and dominance model is

$$\begin{aligned} y_i = \mu + x_i^l \theta _a^l+ w_i^l \theta _d^l +A_i+D_i + e_i \quad \text {(AD model)} \end{aligned}$$

$x_i^l$ is the centered genotype of the *i*th individual at the *l*th marker locus; $\theta _a^l$ is the additive effect of the *l*th locus; $w_i^l$ is defined later; $\theta _d^l$ is the dominance effect of the *l*th locus; $A_i$ is the random additive effect *i*; $D_i$ is the random dominant effect *i*; and $e_i$ denotes error.

Let $\varvec{A}$, $\varvec{D}$, and $\varvec{e}$ denote vectors ($A_i$, $i=1,\ldots ,n$), ($D_i$, $i=1,\ldots ,n$), and ($e_i$, $i=1,\ldots ,n$), respectively, with *n* denotes the number of individuals.

Then $\varvec{A} \sim \mathcal {N}(0,\sigma _a^2\varvec{K_a})$, $\varvec{D} \sim \mathcal {N}(0,\sigma ^2_d\varvec{K_d})$, $\varvec{e} \sim \mathcal {N}(0,\sigma ^2_e\varvec{Id})$, where $\varvec{K_a}$ is the additive kinship matrix; $\varvec{K_d}$ is the dominance kinship matrix; and $\sigma _a^2$, $\sigma _d^2$ and $\sigma ^2_e$ are additive, dominance, and residual variances, respectively. $\varvec{K_a}$ has been defined in the additive model, and $\varvec{K_d} =\varvec{WW'}$ where $$\varvec{W} =\left[ w_i^l\right] _{\begin{array}{c} l=1,\ldots ,L \\ i=1,\ldots ,n \end{array}}$$ with *L* the number of loci; and

1 $$\begin{aligned} w_i^l= \left\{ \begin{array}{ll} -p_1^lp_0^l &{} \text {if the } i\text {th individual is homozygote for the elite allele in the (elite x exotic) progeny,}\\ &{} \text {or for one allele in the elite panel at locus } l \\ 2p_2^lp_0^l &{} \text {if the } i\text {th individual is heterozygote at locus } l \\ -p_2^lp_1^l &{} \text {if the } i\text {th individual is homozyogote for the exotic allele in the (elite x exotic) progeny,} \\ &{} \text {or for the other allele in the elite panel at locus }l \end{array}\right. \end{aligned}$$

where $p_0^l$, $p_1^l$, and $p_2^l$ are the genotypic frequencies for the genotypes 0, 1, and 2 at the locus *l*. It is equivalent to the unscaled formula described in Vitezica et al. (2017) using the NOIA model (Álvarez-Castro and Carlborg 2007).

For the elite panel the structure of the population is also considered, so the model used is:

$$\begin{aligned} y_i = \mu + c_i + x_i^l \theta _a^l+ w_i^l \theta _d^l +A_i+D_i + e_i\quad \text {(AD model with structure)} \end{aligned}$$

where $c_i$ is the cluster to which the *i*th individual belongs.

#### Model selection and SNP estimation

The more integrated the regressors in the models, the lower the remaining trait variance to explain. However, the last SNPs added into the model may have a very small effect, whereas the purpose of GWAS analysis is to find SNPs with strong effects. That is why a parsimony criterion is used to select the best model, where the fewest SNPs explain most of the trait variability. The BIC Bayesian Information Criterion (BIC) is used to find the best model in the elite panel GWAS (2,101 individuals for almost 624 SNPs). However, this criterion is not strict enough for model selection in large model space (Chen and Chen 2008) as in the progeny (only 186 individuals for 604 SNPs). Accordingly, the extended Bayesian Information Criterion (eBIC) (Chen and Chen 2008) was used. It penalizes the BIC calculation according to the number of possible models for a given number of regressors using a mathematical combination. The effects of SNPs selected by eBIC were computed in the AD model, the most complete model, at the best step. Tukey’s test of mean comparison was then performed to analyze the significance of the difference among the three genotypic classes (00 homozygous, 01 or 10 heterozygous, 11 homozygous).

#### QTLs merging

To compare GWAS results in the progeny and in the elite panel, all detected SNPs in a population were merged into QTLs. A QTL was defined as a group of SNPs associated with traits of interest, located on the same chromosome with a maximum of 5 cM between two consecutive SNPs, and with linkage disequilibrium greater than the significance threshold. The significance level of linkage disequilibrium was studied independently for both populations. It was obtained by taking a random sample of 10,000 pairs of markers belonging to different chromosomes, calculating the squared Pearson’s correlation $r^2$ corrected by kinship for the progeny and by the structure for the elite panel (Mangin et al. 2012) between each pair, and taking the $99\%$ quantile of the 10,000 distribution as the threshold. We therefore obtained thresholds of 0.33 and 0.12 for the progeny and for the elite panel, respectively.

## Results

### Phenotypic data analysis

#### (elite $\times$ exotic) progeny

**Fig. 1:**
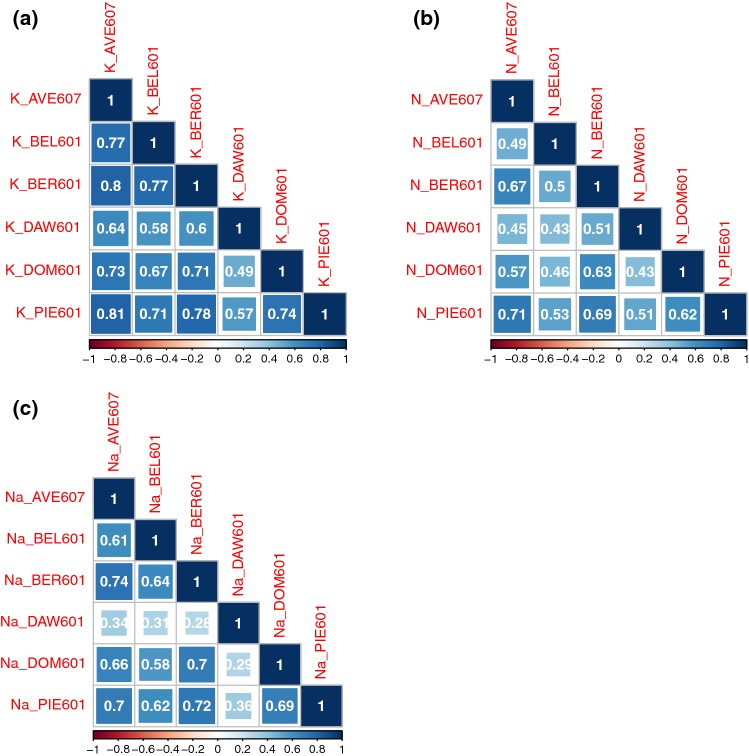
Correlation of potassium content (K; meq/100 g), $\alpha$-amino nitrogen content (N; meq/100 g) and sodium content (Na; meq/100 g) between six consistent environments AVE607, BEL601, BER601, DAW601, DOM601 and PIE601 of (elite $\times$ exotic) progeny. **a** Correlation of potassium content (K; meq/100 g) between six consistent environments of the (elite $\times$ exotic) progeny, **b** Correlation of $\alpha$-amino nitrogen content (N; meq/100 g) between six consistent environments of the (elite $\times$ exotic) progeny, **c** Correlation of sodium content (Na; meq/100 g) between six consistent environments of the (elite $\times$ exotic) progeny

Figure 1 shows correlations between the six environments of the (elite $\times$ exotic) progeny for each of the three impurity traits: potassium content, $\alpha$-amino nitrogen content, and sodium content. Larger and darker squares between two environments indicate a greater positive correlation between the two environments. Larger and redder square between two environments indicates a greater negative correlation between the two environments. All these environments were located in the north of France except DAW601, located in the south of Great Britain. Five of the six environments, AVE607, BEL601, BER601, DOM601, and PIE601, were well correlated for potassium content with correlations values from 0.67 to 0.81 (Fig. 1a). Correlations between these environments and DAW601 were slightly lower but still positive (from 0.49 to 0.64). For the $\alpha$-amino nitrogen (Fig. 1b), the six environments were correlated, but with lower correlation values (from 0.43 to 0.71). AVE607, BER601, DOM601, and PIE601 were well correlated for sodium content with correlation values from 0.66 to 0.74 (Fig. 1c). Correlations between these environments and BEL601 were a little bit lower (from 0.58 to 0.64), and correlations between all these environments and DAW601 were really lower (from 0.28 to 0.36).

Table 1 provides the part of the phenotype variance explained by the genotype for each of the three impurity traits, also called the heritability of the trait. Heritability ranged from 0.44 to 0.85 for the potassium quantity, 0.37 to 0.80 for the sodium quantity, and from 0.45 to 0.75 for the $\alpha$-amino nitrogen. The heritability of the three traits was lower in DAW601 than in other environments. This environment was in Great Britain, whereas all the others were in France; thus, its soil could be rather different from that of the others. The heritability of sodium content and $\alpha$-amino nitrogen content were also quite low in BEL601.

**Table 1:** Heritabilities ($h^2$) in each of the six consistent environments of the (elite $\times$ exotic) progeny (AVE607, BEL601, BER601, DAW601, DOM601 and PIE601) for potassium content (K; meq/100 g), sodium content (Na; meq/100 g), and $\alpha$-amino nitrogen content (N; meq/100 g)

	AVE607	BEL601	BER601	DAW601	DOM601	PIE601
$h^2_K$	0.82	0.75	0.87	0.44	0.74	0.85
$h^2_{Na}$	0.76	0.59	0.77	0.37	0.75	0.80
$h^2_N$	0.68	0.49	0.73	0.45	0.63	0.75

#### Elite panel

Table 2 shows heritabilities of each of the three impurity traits in the elite panel and for each of its clusters. Heritability calculated for each trait in the entire panel was similar to the higher heritability found in the progeny for the corresponding traits.

**Table 2:** Heritabilities ($h^2$) in each panel and in the entire population of the elite panel for potassium content (K; meq/100 g), sodium content (Na; meq/100 g), and $\alpha$-amino nitrogen content (N; meq/100 g)

	Entire panel	Panel A	Panel B
$h^2_K$	0.88	0.68	0.84
$h^2_{Na}$	0.77	0.42	0.73
$h^2_N$	0.70	0.42	0.62

### Mapping

There were 604 distinct SNPs for the progeny; 448 were mapped. There were 626 distinct SNPs for the elite panel; 322 were mapped.

The information regarding the genetical maps of population used to create the consensus map and the genetical consensus map is given in Tables 3 and 4, respectively. The standard nomenclature of the nine chromosomes of sugar beet (Butterfass 1964) is used.

**Table 3:** Number of SNPs and length of each chromosome of the four genetic maps used to create the consensus map (cM: Haldane)

	Population 804		Population 805		Population 809		Population 813	
	SNPs	Length (cM)	SNPs	Length (cM)	SNPs	length (cM)	SNPs	Length (cM)
Chromosome 1	934	70.9	863	79.4	846	66.5	314	83.2
Chromosome 2	1184	76.8	819	77.1	683	77.4	624	86.4
Chromosome 3	1525	69.8	1115	90.6	1005	94.5	1356	103.4
Chromosome 4	1124	75.7	972	77.2	998	99.3	1097	114.2
Chromosome 5	97	10.9	1101	73.7	883	72.6	–	–
Chromosome 6	–	–	902	97.2	587	84.1	850	108.5
Chromosome 7	13	1.3	508	75.8	817	94.6	102	50.6
Chromosome 8	1411	91.2	1302	87.6	1191	113.1	–	–
Chromosome 9	80	25.4	1005	90.3	964	113.4	900	139.1
Total	6368	422	8587	748.9	7974	815.5	6445	828.4

The four genetic maps were created from four (elite $\times$ exotic) populations generated in the AKER project

**Table 4:** Number of SNPs and length of each chromosome of the genetic consensus map (cM: Haldane) 91 SNPs were then added in the consensus map, from the propriety map

	SNPs	Length (cM)
Chromosome 1	1054	76
Chromosome 2	1205	84.2
Chromosome 3	1637	89.7
Chromosome 4	1290	91.6
Chromosome 5	1130	73.7
Chromosome 6	941	96.6
Chromosome 7	844	73.1
Chromosome 8	1597	123.4
Chromosome 9	1140	116.2
Total	10,838	824.5

From 0 to 31 SNPs were added to each chromosome, with a mean of 13

### QTL detection results

#### (elite $\times$ exotic) progeny

Association studies with the A model and with the AD model were performed on the six environments of the (elite $\times$ exotic) progeny and on the mean phenotype for each of the three impurity traits: potassium content, $\alpha$-amino nitrogen content, and sodium content. As the mean phenotype is certainly the most interesting trait for breeding, we first present results on this mean phenotype. After filtration with the eBIC criterion, 16 distinct SNPs were detected. All were detected with the A model, except one which is only detected with the AD model. Another one was detected with both models. As the studied traits were impurities, we can say that the exotic allele of a detected SNP had a favorable effect if the trait value decreased in the presence of this exotic allele. Table 5 provides details about all SNPs detected with the mean phenotypes.

**Table 5:** SNPs associated with potassium content (K; meq/100 g), sodium content (Na;meq/100 g), and $\alpha$-amino nitrogen content (N; meq/100 g) for the mean phenotype of (elite $\times$ exotic) progeny

SNP	Trait	Model	Chr	Position	%var	Favorable.exotic
SNP_10753	Na	A	9	105.93	0.11	Yes
SNP_06641	Na	AD	6	31.43	0.13	No
SNP_07975	K	A	7	66.69	0.18	No
SNP_07975	K	AD	7	66.69	0.18	No
SNP_06319	N	A	5	64.98	0.52	No
SNP_00322	Na	A	1	46.05	0.20	No
SNP_06273	Na	A	5	56.20	0.05	No
SNP_01689	Na	A	2	22.23	0.02	Yes
SNP_05508	Na	A	5	30.14	0.07	No
SNP_00116	Na	A	1	21.86	0.08	Yes
SNP_02804	Na	A	3	50.78	0.04	Yes
SNP_00350	Na	A	1	51.00	0.04	Yes
SNP_09633	Na	A	8	95.03	0.06	Yes
SNP_09271	Na	A	8	67.17	0.04	No
SNP_09818	Na	A	9	10.12	0.06	Yes
SNP_09973	Na	A	9	36.71	0.05	Yes
SNP_06344	N	A	5	68.13	0.18	Yes

These SNPs are detected in association studies with an additive model (A) and an additive and dominance model (AD), and selected with the eBIC criterion. Their position on chromosome, the proportion of variance they explained in the multi SNPs model selected by eBIC (%var), and information about the favorable or unfavorable effect of the exotic allele are also given

**Fig. 2:**
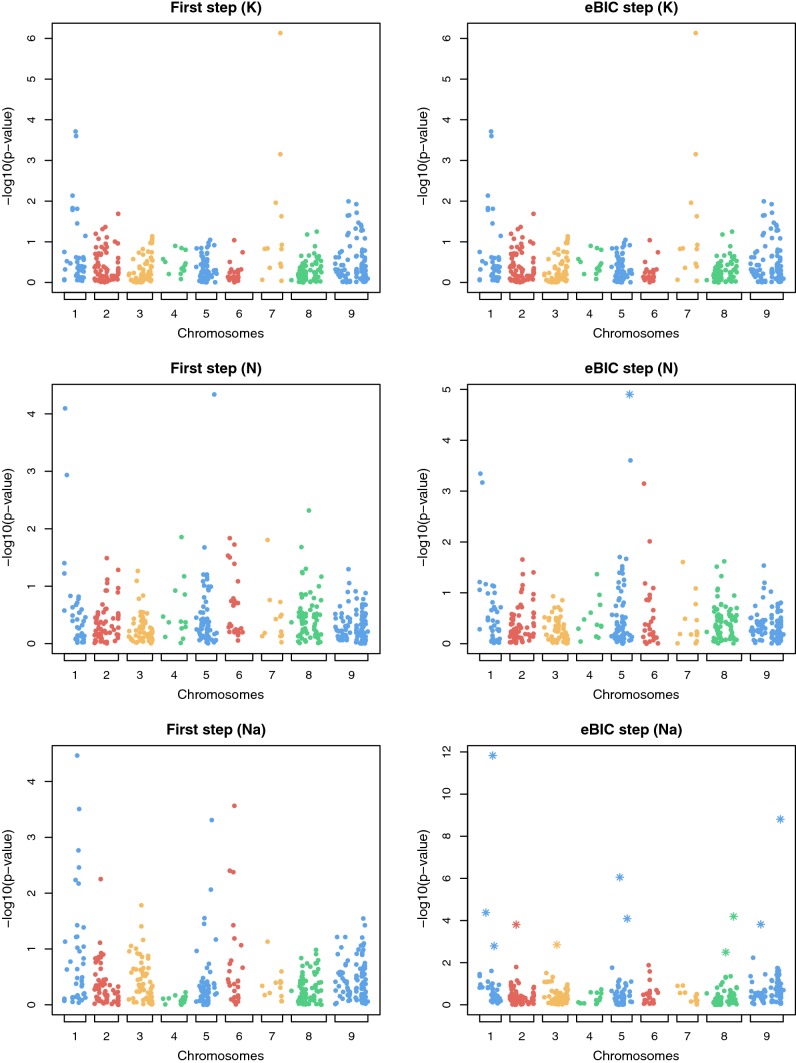
Manhattan plots in the (elite $\times$ exotic) progeny using the additive model of the first step of GWAS on the left, and the step selected by eBIC on the right for the mean phenotype of potassium content on the first row, for the mean phenotype of $\alpha$-amino nitrogen content on the second row and for the mean phenotype of sodium content on the third row. Note that the two steps can be the same. Stars in the step selected by eBIC represent SNPs detected and added into the model in previous steps

In Fig. 2 QTL detection results are illustrated with Manhattan plots using the A model of the first GWAS forward step, which is the usual GWAS analysis, and the GWAS forward step selected by eBIC for the mean phenotype of each impurity traits in the (elite $\times$ exotic) progeny. Note that the two steps could be the same. These Manhattan plots on all environments of the (elite $\times$ exotic) progeny are given in supplementary material (see in supplementary material Figs. S9–S14).

**Fig. 3:**
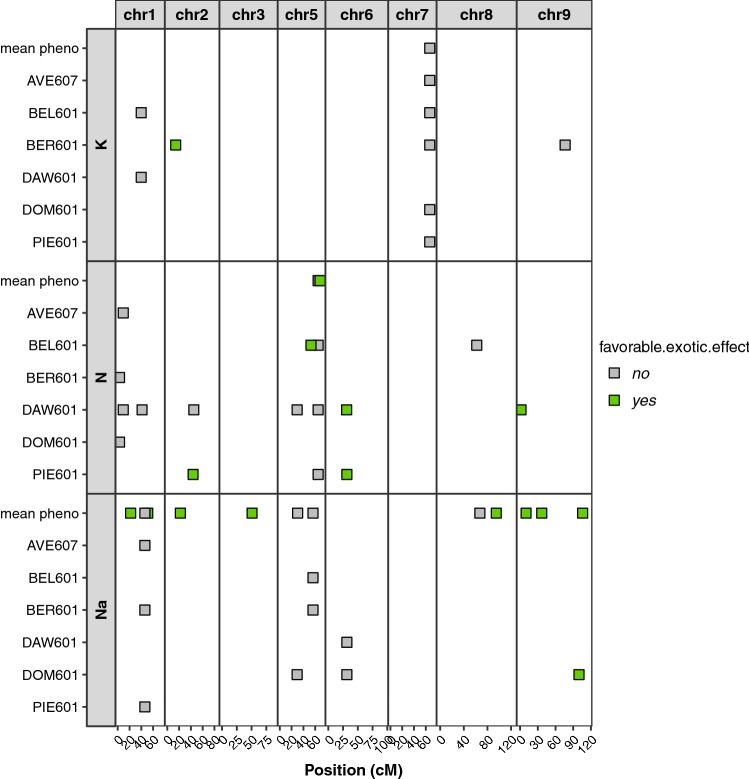
SNPs detected in the (elite $\times$ exotic) progeny for potassium content (K, meq/100 g), sodium content (Na; meq/100 g) and $\alpha$-amino nitrogen (N; meq/100 g) with the mean phenotype and in each environment

We wanted to see whether detected SNPs in the mean phenotype were also detected in the six environments. After filtration with the eBIC criterion, 33 distinct SNPs were detected in total on the six environments and the mean phenotype. All were detected with the A model, and three were also detected with the AD model. Figure 3 shows all of them in the (elite $\times$ exotic) progeny.

Four SNPs associated with the potassium content were detected. One SNP on chromosome 7 was found in all environments, except in DAW601, whereas the others were detected in one or two environments. This SNP was also found with the AD model in four environments. Another SNP found on the chromosome 2 in BER601 was the only to have a favorable exotic allele effect. A total of 14 SNPs associated with $\alpha$-amino nitrogen content were detected by the A model, and one SNP_00018, was also found with the AD model. Three of these detected SNPs were detected in two or more environments, including SNP_00018. Six SNPs only detected in one environment had a favorable exotic allele. One SNP was not mapped. A total of 16 SNPs were detected for the sodium content with the A model, most of them for the mean phenotype. One of them was also found with the AD model. Nine had a favorable exotic allele. One SNP was not mapped. There was one SNP in common for $\alpha$-amino nitrogen content and sodium content on chromosome 6 (see Venn diagram in supplementary material Fig. S15) not detected in the same environment for both traits.

Detected SNPs were then merged into QTLs. A QTL grouped together SNPs that were on the same chromosome, with no more than 5 cM between two consecutive SNPs, and with linkage disequilibrium greater than the predefined significance threshold (0.33 for the progeny). If a detected SNP cannot be merged with another, it alone represented a QTL. For the 33 distinct detected SNPs, 31 were mapped on the consensus map; one SNP associated with the $\alpha$-amino nitrogen content and one linked with the sodium content were not mapped. In the progeny, 30 QTLs have been defined. Each SNP not mapped constituted a QTL. Others were also composed of only one SNP, except one which merged 2 SNPs on chromosome 5. The list of all SNPs associated with potassium content, $\alpha$-amino nitrogen, and sodium content, with the name of the QTL to which they belong, the environment in which they were detected, the model used, the chromosome on which they were located, their position on this chromosome, the part of the phenotype variance they explained, and whether they had a favorable effect of the exotic allele are presented in the supplementary material (see in supplementary material Tables S1, S2, S3).

#### Elite panel

Association studies were performed for each of the three impurity traits with the A model and with the AD model on the elite panel, and on each of its two clusters (Panel A with 676 individuals, and Panel B with 1425 individuals). Table 6 lists the number of detected SNPs detected only with the A model, only with the AD model or with both models for each of the three impurity traits after the selection by eBIC criterion.

**Table 6:** Number of detected and SNPs for potassium content (K; meq/100 g), sodium content (Na; meq/100 g), and $\alpha$-amino nitrogen content (N; meq/100 g) in elite panel, with the additive model (A) and the additive and dominance model (AD) or in both models

Trait	A model only	AD model only	Both models
K	60	0	5
N	40	2	0
Na	75	3	9

The majority of SNPs were detected using the A model. The AD model added only five new SNPs. Fourteen were found by both models. Several SNPs were found in common between two or three traits (see the Venn diagram in supplementary material Fig. S16), but none were detected by the AD model only. Finally, a total of 177 distinct SNPs were detected for all traits. Association study results on the entire panel, the panel A and the panel B were illustrated with Manhattan plots (see in supplementary material Figs. S18 to S20).

Detected SNPs were then merged into QTLs. A QTL groups together SNPs that are on the same chromosome, with no more than 5 cM between two consecutive SNPs, and with linkage disequilibrium greater than the predefined significance threshold (0.12 for the elite panel). If a detected SNP cannot be merged with another, it alone represented a QTL. Of the 177 distinct detected SNPs, 171 were mapped on the consensus map. One SNP detected associated with the potassium content, one linked with $\alpha$-amino nitrogen content, and three linked with sodium content were not mapped. In the elite panel, 97 QTLs were defined. Each unmapped SNP constituted a QTL, and 35 other QTLs were also composed by only one SNP, and there were therefore 40 QTLs with only one SNP. Fifty-seven QTLs merged two SNPs or more and the three largest were composed of eight SNPs. Two of them were located on chromosome 1, and the other was on chromosome 7. The list of all SNPs detected that were associated with potassium content, $\alpha$-amino nitrogen, and sodium content, with the name of the QTL to which they belong, the environment in which they were detected, the model used, the chromosome on which they were located, their position on this chromosome, the part of the phenotype variance they explained, and whether they had a favorable effect of the exotic allele are presented in the supplementary material (see in supplementary material Tables S4, S5, S6).

### QTL mapping

**Fig. 4:**
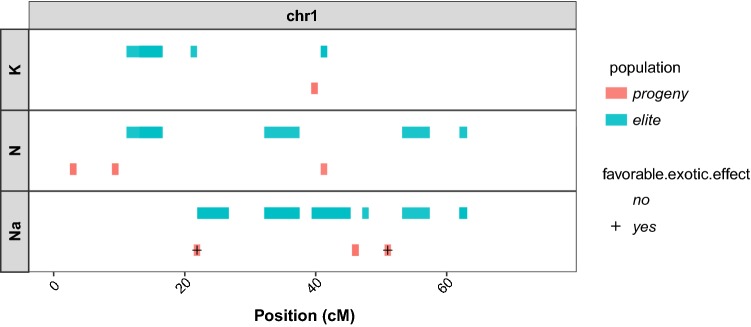
QTLs detected in (elite $\times$ exotic) progeny and elite panel for potassium content (K; meq/100 g), sodium content (Na; meq/100 g), and $\alpha$-amino nitrogen content (N; meq/100 g) mapped on chromosome 1 of the consensus map. The favorable effect of an exotic allele is indicated by the ’+’ sign

Figure 4 shows QTLs detected in the mean phenotype on chromosome 1 in (elite $\times$ exotic) progeny and in elite panel populations for each impurity trait. As the studied traits were impurities, the effects of the exotic allele was considered favorable when it was associated with a decrease in the amount of the impurity. We found more QTLs in the elite panel than in the (elite $\times$ exotic) progeny. Some QTLs found in the (elite $\times$ exotic) progeny were very close or collocated with those of the elite panel and could have a favorable effect on the exotic allele, as the first QTLs detected that were associated with sodium content, or an unfavorable effect of the exotic allele, as the second QTL detected that had an association with sodium content and the QTL detected that were associated with potassium content. Other QTLs of the (elite $\times$ exotic) progeny were not collocated with QTLs detected in the elite panel. These QTLs could also have a favorable effect on the exotic allele, as the third QTL detected that was associated with sodium content, or an unfavorable effect of the exotic allele, as the three QTLs detected that were associated with $\alpha$-amino nitrogen content. However, if we look at the coverage of the chromosome with the elite SNPs (see in the supplementary material Fig. S8b), we can see that there was no elite SNP in the region with the first two QTLs associated with $\alpha$-amino nitrogen content in (elite $\times$ exotic) progeny. Thus, with elite SNPs covering this region, we would have also detected it in the elite panel. Results on each chromosome are given in supplementary material (see in supplementary material Fig. S17). Table 7 gives all QTLs detected in the mean phenotype in the (elite x exotic) progeny that had a favorable exotic effect.

**Table 7:** SNPs associated with the mean phenotype for potassium content (K; meq/100 g), sodium content (Na; meq/100 g), and $\alpha$-amino nitrogen content (N; meq/100 g) of (elite $\times$ exotic) progeny, which have favorable effect of the exotic allele

SNP	QTL	Trait	Model	Chr	Position	%var	Favorable exotic
SNP_00116	QTL_19_1	Na	A	1	21.86	0.08	Yes
SNP_00350	QTL_09_1	Na	A	1	51.00	0.04	Yes
SNP_01689	QTL_18_1	Na	A	2	22.23	0.02	Yes
SNP_02804	QTL_07_1	Na	A	3	50.78	0.04	Yes
SNP_06344	QTL_43_1	N	A	5	68.13	0.18	Yes
SNP_09633	QTL_12_1	Na	A	8	95.03	0.06	Yes
SNP_09818	QTL_30_1	Na	A	9	10.12	0.06	Yes
SNP_09973	QTL_35_1	Na	A	9	36.71	0.05	Yes
SNP_10753	QTL_29_1	Na	A	9	105.93	0.11	Yes

These SNPs are detected in association studies with an additive model (A) and an additive and dominance model (AD), selected with the eBIC criterion and merged into QTLs. Their position on chromosome and the proportion of variance they explained in the multi SNPs model selected by eBIC (%var) are also given

## Discussion

In this work, we compared the detected QTLs found for three impurities traits in two different populations, an (elite $\times$ exotic) progeny composed of 187 individuals and an elite panel composed of 2101 individuals.

The elite panel was divided into two panels by hierarchical clustering. Panel A and panel B contained 676 and 1425 individuals, respectively, so one-third for panel A and two-third for panel B. The cause of this structure was not known, but it had an impact on heritabilities calculated for the three impurity traits in these two panels: heritabilities were one-third lower in panel A. This population was already studied for genomic prediction in Mangin et al. (2019) where authors showed that the accuracy of prediction in panel B decreases when individuals from panel A are added, whereas a decrease in the accuracy of prediction for panel A is not observed. This is probably due to the larger size of the panel A. Both heritabilities and genomic prediction suggested that the two panels were different, so QTL detection was performed on each of these panels in addition to QTL detection on the full elite panel. Moreover, even we do not have the precise pedigree we know that the elite panel is composed by many biparental populations. Maybe founders of theses populations are very different between the two panels.

Because the individuals from the progeny were related, we could have used a linkage mapping analysis rather than association mapping for QTL detection in this population. To understand why we can use these two methods interchangeably, it is essential to understand what distinguishes them. Linkage analysis and association analysis are both based on linear models, with a statistical test at each position of a putative QTL that gives a scan on the whole genome. The main difference is that linkage analysis makes a test not only at the markers but also between the markers. Another difference is the use of fixed effect model for linkage analysis and mixed model for association analysis. However, mixed model have already been proposed for linkage analysis. They were first used in human pedigrees by Pratt et al. (2000) who assumed that the QTL effect was random. Then, for simple pedigree Pérez-Enciso and Varona (2000) proposed a mixed model of the QTL effect with a fixed part tested at each locus of the chromosome and a random part that considers the relationship between individuals. This model for linkage analysis is identical to that of association analysis. The test performed on the markers by the two methods is also similar if the polygenic variance included in the association model is very low. This follows from the analytical formula of the Wald test used for the association method. This decrease in polygenic variance is obtained by the forward selection procedure of MLMM (Segura et al. 2012). Indeed, at each step of MLMM, the marker that is the most associated with the trait is added in the association model as a regressor and the polygenic variance decreases. Finally, there is no detectable QTL in the polygenic effect of the association model; they are all fixed effects in the model, similar to the case of linkage analysis with cofactors. Moreover, the choice of cofactors in the linkage analysis is treated in a similar way, because a similar forward procedure limited to markers has been proposed by Jourjon et al. (2005) to decide which cofactors to include in the linkage analysis model. These two methods are thus a response to a model with several QTLs. This is also discussed in Bonnafous et al. (2018), who argues that because the location of multiple QTLs is unknown the association analysis model assumes that each marker is a QTL. The addition of a law on the effects of markers then results in a polygenic effect with a matrix of relatedness, also called kinship. This kinship should be different for each locus, which would increase the power of the association model (Rincent 2014), but for computational cost reasons, generally the same matrix is used for the entire genome. As we have just seen, the tests of the two methods are similar with regard to the markers, but in the linkage analysis pseudo-markers are also used. Rebai et al. (1995) have shown that these pseudo-markers provide only 5% more power compared to a marker per marker analysis when the distance between two markers is less than 20 cM. In the (elite $\times$ exotic) progeny, the two most distant markers were spaced apart by 11.26 cM (markers located on chromosome 8). Testing the QTLs only on markers does not therefore lose a lot of detection power. Finally, because the identity by state (IBS) and the identity by descent (IBD) are identical in a progeny, an association model using an IBS type kinship uses the Mendelian allelic transmission.

Two models were used to do the QTL detection: an additive model and additive and dominance model. Note that the above models are statistical not genetical. For the A model and a testcross, the descendant mean of pollinisators that are heterozygous at a marker is assumed to be the average of the descendant mean of the two homozygous pollinisators. For the AD model, this assumption is not made leading to one more fixed parameter and one more variance component to estimate. These additional parameters cause a decrease in power compared to the A model if the actual dominant effect is not substantial. Most GWAS models consider only the additive effect of markers. However, several studies have shown that the non-additive effects constitute a major part of the variation of complex traits. Bonnafous et al. (2018) showed, by studying the phenotypic hybrid value, that the AD model makes it possible to find SNPs with a dominant effect. Indeed, with hybrids, the AD model corresponds to the model with a genetically dominance. However as we have testcross values, we cannot conclude on the genetically dominance of detected SNPs. Moreover the statistical dominance modeling in the progeny can be proved to be linked to regions with segregation distortion by using the usual decomposition with genetically additive and dominance effects. Besides in the (elite $\times$ exotic) progeny none SNPs were detected with the AD model only, so we could only have used the A model for the QTL detection.

Many more QTLs have been detected in the elite panel than in the progeny (87 and 33, respectively). On several chromosomes, the only QTLs detected were found in the elite panel, and when QTLs were found in the (elite $\times$ exotic) progeny, they were often also found in the panel. This may be because genetic diversity was much more important in the elite panel than in the progeny, since the (elite $\times$ exotic) progeny was a biparental population whereas the elite panel was composed by a lot of small biparental populations. Moreover, the elite panel was composed of 2101 individuals, whereas the (elite $\times$ exotic) progeny was only composed of 187 individuals. Because of this difference in size between the two populations, there was more statistical power in the elite panel for the QTL detection than in the (elite $\times$ exotic) progeny.

On the 33 QTLs detected in the (elite $\times$ exotic) progeny for all impurity traits, only 16 had a favorable exotic allele. The majority of favorable alleles were therefore also present in the elite parent. This was expected as the elite parent was the result of an artificial selection by breeders in the Florimond Desprez company. The sodium content, the $\alpha$-amino nitrogen content, and the potassium content were well-measured traits with high heritability, so the selection process has probably already fixed the favorable QTL alleles. In addition, the selection of sugar beet is based on the white sugar yield and, the calculation of the white sugar yield being a function of the impurity content, this choice led to the recruitment of alleles favorable to the reduction of impurities, although selective scanning is currently lacking in sugar beet Adetunji et al. (2014).

**Fig. 5:**
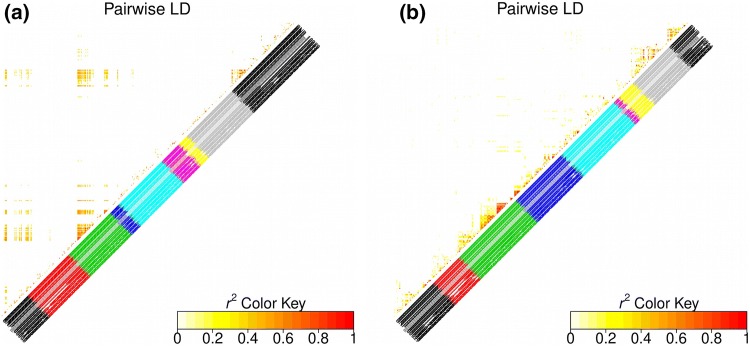
Heatmap of $r^{2}$ values between all possible pairs of mapped SNPs in both populations. **a** LD in the progeny, corrected for genetic relatedness, **b** LD in the elite panel, corrected for the structure in two clusters

Because of the sugar beet breeding history, we know that all chromosomes have not been subjected to the same selection intensity. For example, the heatmap of the progeny shows a strong LD between chromosome 3 and chromosome 9 (Fig. 5a), which is possibly the result of selection for rhizomania resistance. The *Rz*1 on chromosome 3 comes from a single source and its introgression into elite sugar beet breeding lines occurred very rapidly, such that it might have created a genetic bottleneck leading to high LD on other regions of the genome, such as the strong LD on top of chromosome 9 (Adetunji et al. (2014)). We also observed a strong LD between chromosome 3 and chromosome 1, which may also be caused by rhizomania resistance selection. Therefore, SNPs on chromosomes with interchromosomal LD are not independent. The LD threshold in the progeny was probably overestimated. The heatmap of the elite panel did not show interchromosomal LD (Fig. 5b), and there were enough recombination events to break the long range LD. However, we observed several LD intrachromosomes, in particular on chromosome 3, 8, and 9. This high interchromosomal LD could be due to bad mapping of some markers and could cause very long QTLs in the elite panel. To avoid excessively long QTLs, we decided to cut the QTLs into 5 cM intervals in both populations.

Regarding the results, the comparison of the detected QTLs in both populations allowed us to highlight interesting regions in the (elite $\times$ exotic) progeny genome. Indeed, some detected QTLs in the (elite $\times$ exotic) progeny had a favorable exotic allele on different chromosomes because they were linked with a decrease in the impurity content. Some of them were collocated with detected QTLs in the elite panel, others were found in new regions. To go further in the comparison of the progeny and the elite panel, it would have been interesting to included the elite parent of progeny in the elite panel. Moreover, it could have been interesting to study a progeny generated from the same exotic accession but crossed with another elite line. The genetic background could indeed influence allelic fitness (Ungerer et al. 2003) and therefore hide some interesting regions in the (elite $\times$ exotic) progeny. Furthermore, a better genome coverage could have allowed the detection of other QTLs in both populations.

To conclude, the comparison of the detected QTLs in an (elite x exotic) progeny and an elite panel allows the detection of new favorable alleles and genomic regions brought by the exotic accession. Their introgression in a sugar beet elite germplasm is therefore an interesting approach to increasing the genetic diversity that is useful in breeding programs.

### **Author contribution statement**

BM, EG, BruD, KH designed the experiment, PPE, BM, and EG performed all the statistical analyses, KH provided the genetic resources, NH provided the phenotypic data, OG built the genetic map, BriD and PD realized the genotyping of the worldwide population, GW provided the genomic data of the elite population, PPE wrote the draft manuscript with the help of BM, EG, OG, PD, NH and BruD, and all authors read and approved the final manuscript.

## Electronic supplementary material

Below is the link to the electronic supplementary material.

Supplementary material 1 (pdf 2029 KB)
