# Supplementary material for: Discovery of interesting new polymorphisms in a sugar beet (elite × exotic) progeny by comparison with an elite panel
Source: Theor Appl Genet. 2019 Sep 4;132(11):3063–78. doi: 10.1007/s00122-019-03406-0 (PMC6791908; doi:10.1007/s00122-019-03406-0)
Supplement: Supplementary file 1 — Supplementary material 1 (pdf 2029 KB) [file 122_2019_3406_MOESM1_ESM.pdf]

Theoretical and Applied Genetics
https://doi.org/10.1007/s00122-019-03406-0

Discovery of interesting new polymorphisms in a sugar beet (elite x exotic) progeny by comparison with an elite panel

Prune Pégot-Espagnet[1,2] · Olivier Guillaume[1] · Bruno Desprez[2] · Brigitte Devaux[2] · Pierre Devaux[2] · Karine Henry[2] · Nicolas Henry[2] · Glenda Willems[3] · Ellen Goudemand[2] · Brigitte Mangin[1]

Received: 21 December 2018 / Accepted: 24 July 2019

Supplementary material

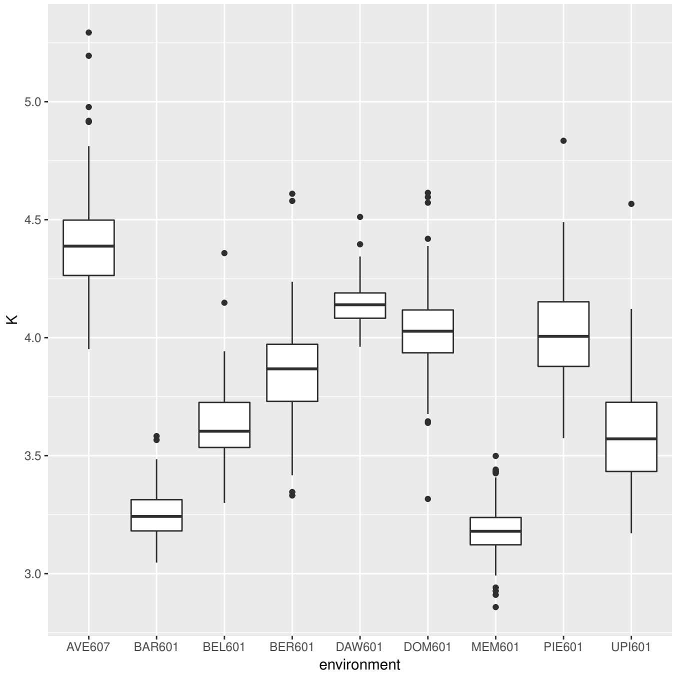

Fig. S1: Boxplots of potassium content (K; meq/100g) in each of the nine environments of the (elite x exotic) progeny after adjustment with SpATS package. The same 187 accessions are present in the 9 environments

Brigitte Mangin
E-mail: brigitte.mangin@inra.fr

[1] LIPM, Université de Toulouse, INRA, CNRS, Castanet-Tolosan, France
[2] Florimond Desprez Veuve & Fils SAS, BP41, 3, Rue Florimond Desprez, Capelle-en-Pévèle 59242, France
[3] SESVanderHave, Industriepark Soldatenplein Zone 2/Nr 15, 3300 Tienen, Belgium

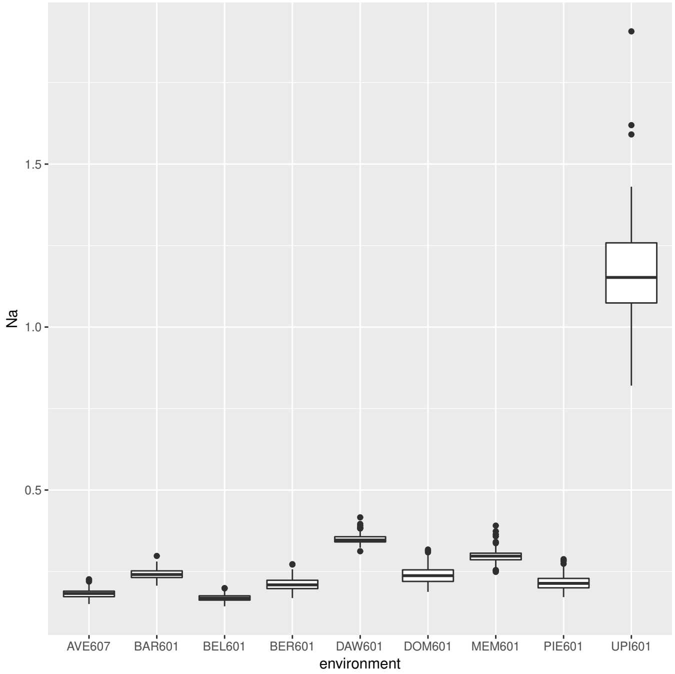

Fig. S2: Boxplots of α-amino nitrogen content (N; meq/100g) in each of the nine environments of the (elite x exotic) progeny after adjustment with SpATS package. The same 187 accessions are present in the 9 environments

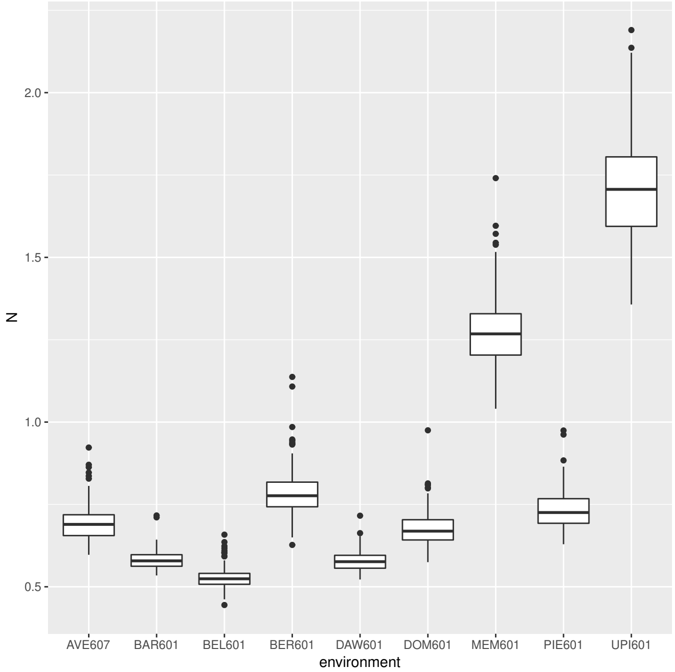

Fig. S3: Boxplots of sodium content (Na; meq/100g) in each of the nine environments of the (elite x exotic) progeny after adjustment with SpATS package. The same 187 accessions are present in the 9 environments

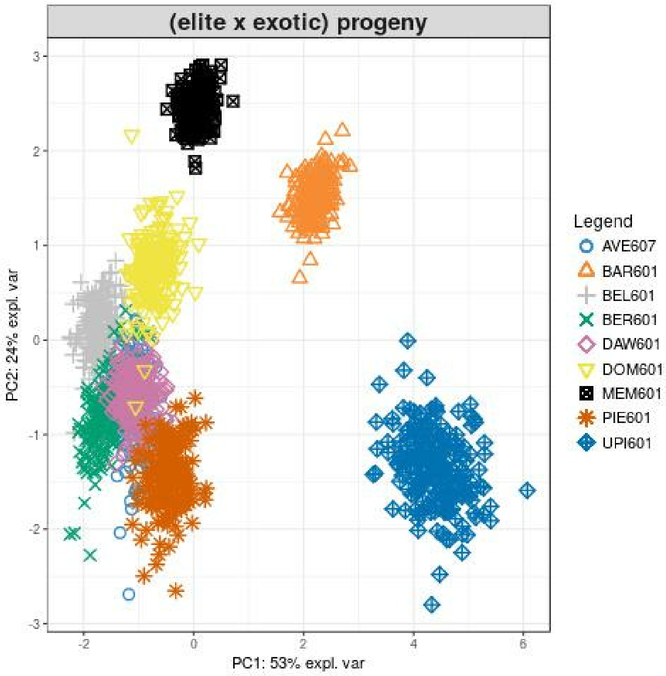

Fig. S4: Principal Component Analysis of the nine environments of the (elite x exotic) progeny according to impurities (potassium content (K; meq/100g), sodium content (Na; meq/100g), and α-amino nitrogen content (N; meq/100g)) and productivity traits (not publicly available). The same 187 accessions are present in the 9 environments

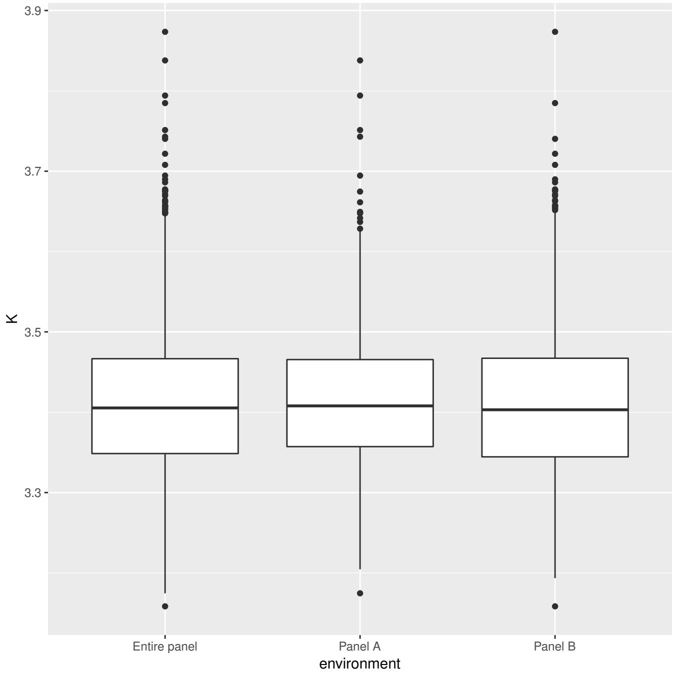

Fig. S5: Boxplots of potassium content (K; meq/100g) in each panel and in the entire elite panel.

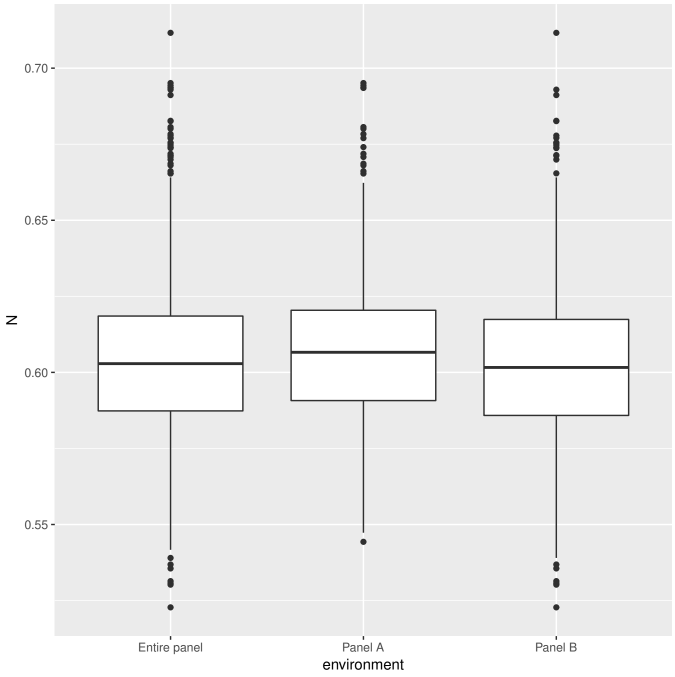

Fig. S6: Boxplots of α-amino nitrogen content (N; meq/100g) in each panel and in the entire elite panel.

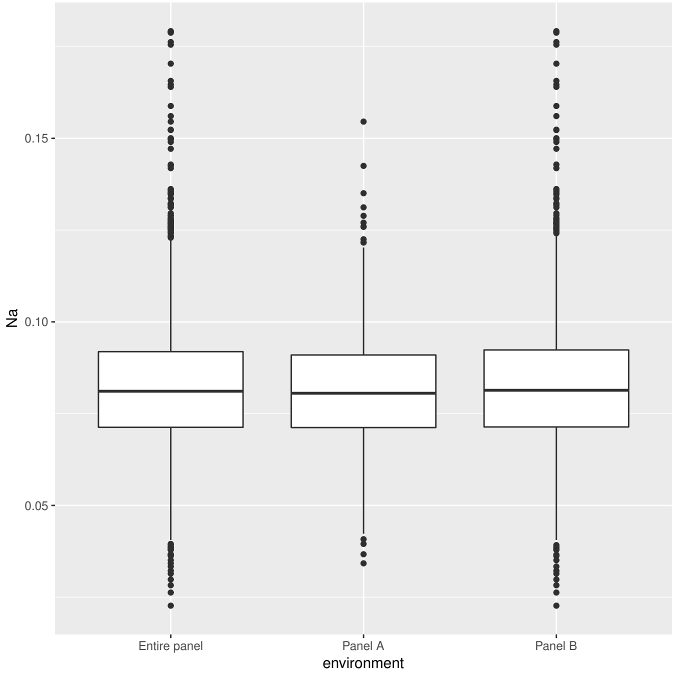

Fig. S7: Boxplots of sodium content (Na; meq/100g) in each panel and in the entire elite panel.

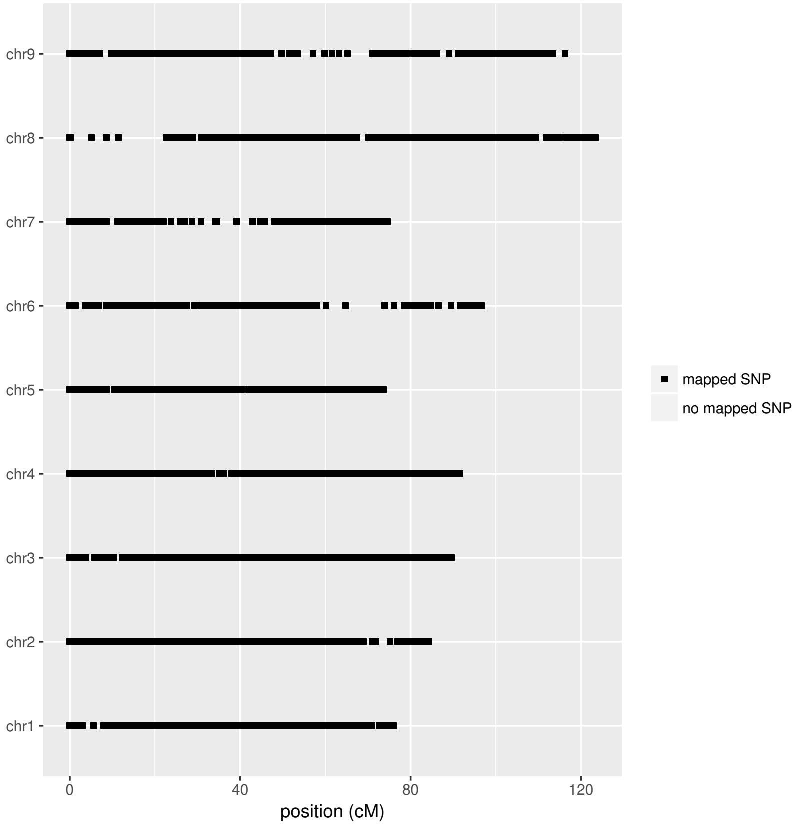

(a) Marker density

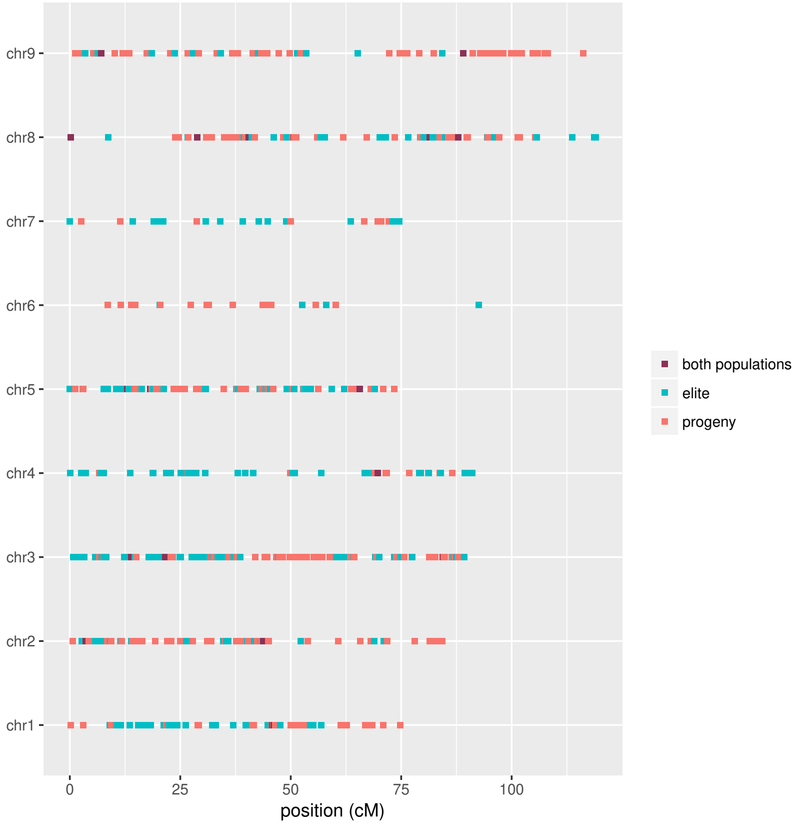

(b) Genotyped and mapped markers in each population

Fig. S8: Marker density: all SNPs present in the consensus map (S8a) and SNPs genotyped in (elite x exotic) progeny, elite panel, or in both populations, mapped on the consensus map (S8b)

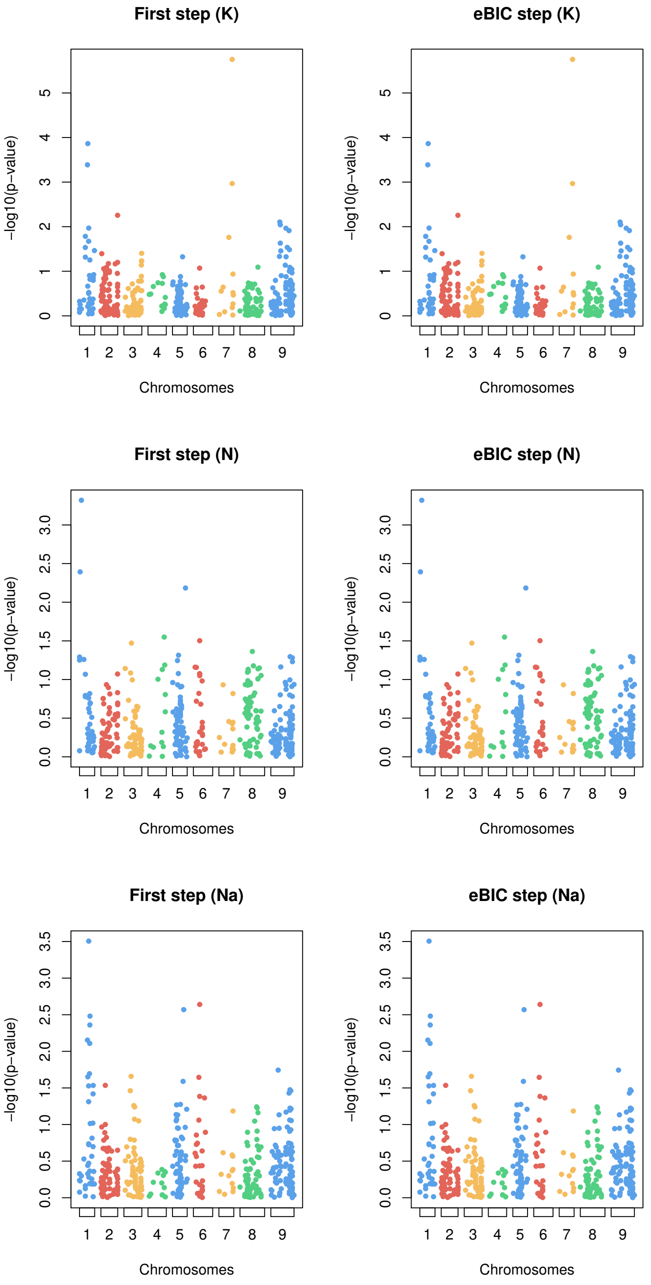

Fig. S9: Manhattan plots using the additive model of the first step of GWAS on the left, and the step selected by eBIC on the right for potassium content in AVE607 of (elite x exotic) progeny on the first row, for α-amino nitrogen content in AVE607 of the (elite x exotic) progeny on the second row and for sodium content in AVE607 of the (elite x exotic) progeny on the third row. Note that the two steps can be the same.

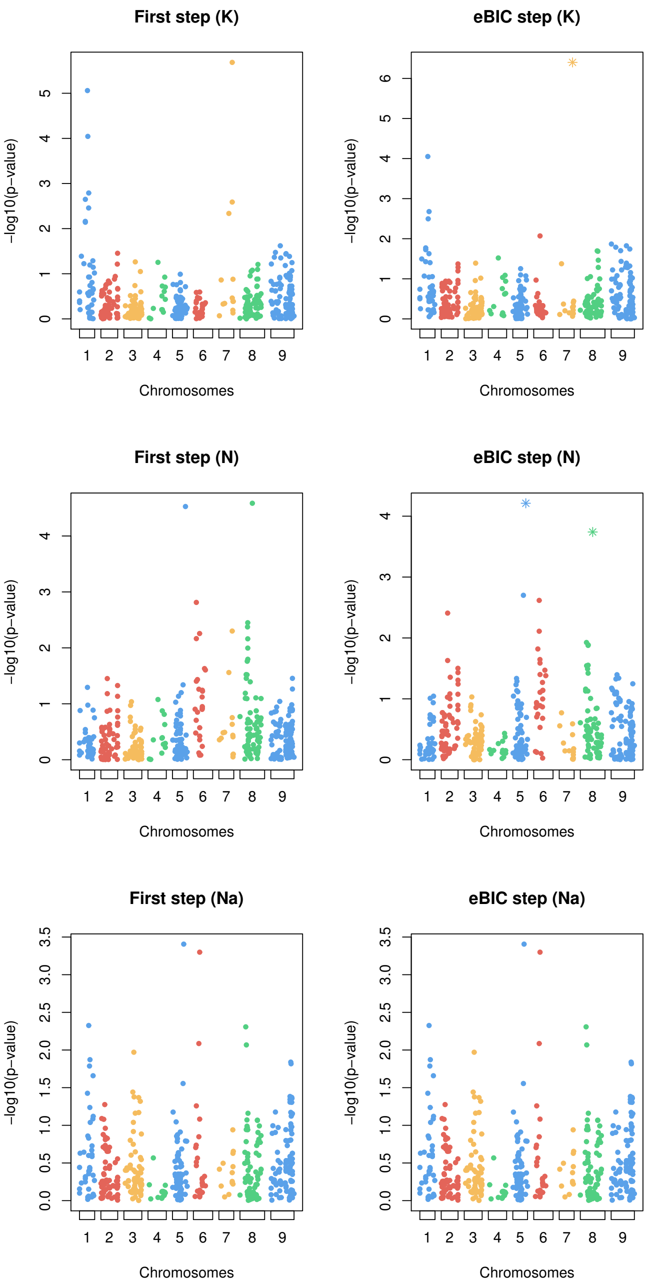

Fig. S10: Manhattan plots using the additive model of the first step of GWAS on the left, and the step selected by eBIC on the right for potassium content in BEL601 of (elite x exotic) progeny on the first row, for α-amino nitrogen content in BEL601 of the (elite x exotic) progeny on the second row and for sodium content in BEL601 of the (elite x exotic) progeny on the third row. Note that the two steps can be the same. Stars in the step selected by eBIC represent SNPs detected and added into the model in previous steps

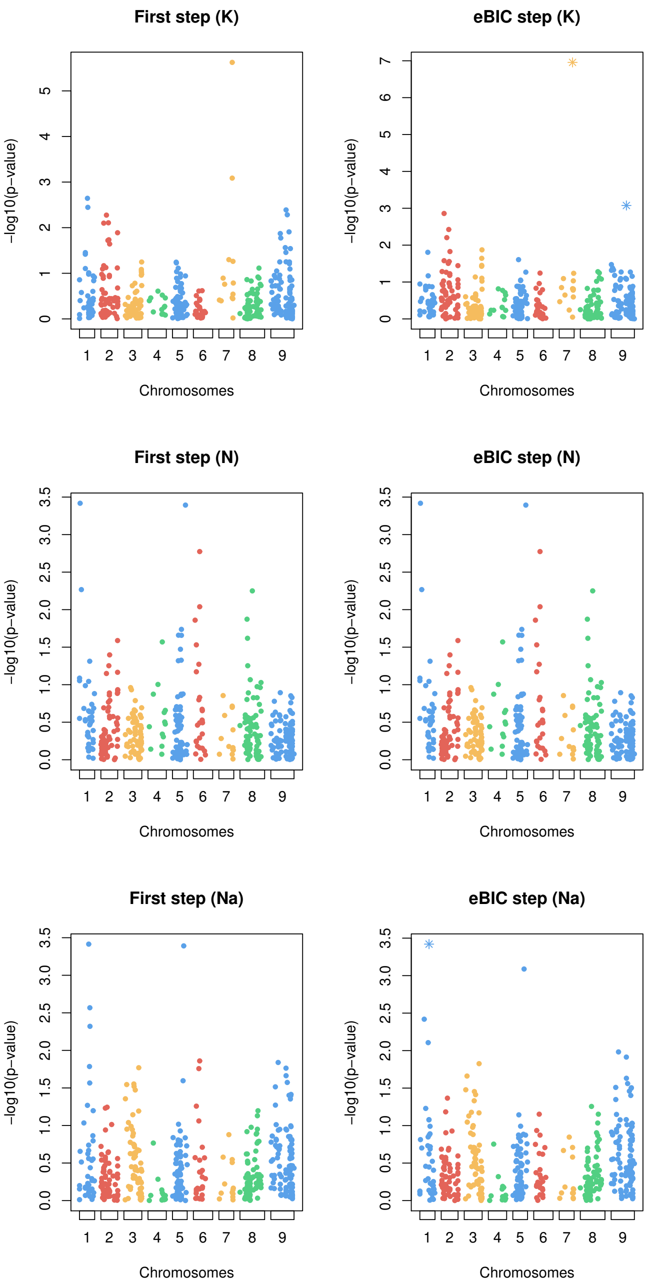

Fig. S11: Manhattan plots using the additive model of the first step of GWAS on the left, and the step selected by eBIC on the right for potassium content in BER601 of (elite x exotic) progeny on the first row, for α-amino nitrogen content in BER601 of the (elite x exotic) progeny on the second row and for sodium content in BER601 of the (elite x exotic) progeny on the third row. Note that the two steps can be the same. Stars in the step selected by eBIC represent SNPs detected and added into the model in previous steps

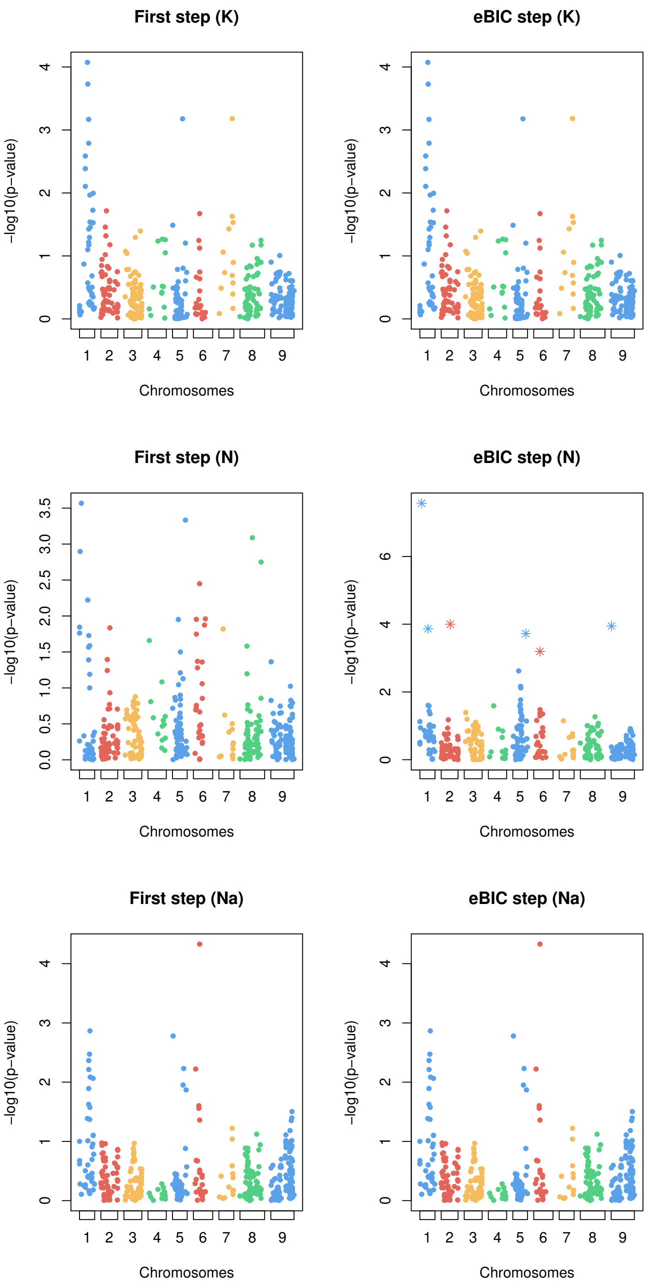

Fig. S12: Manhattan plots using the additive model of the first step of GWAS on the left, and the step selected by eBIC on the right for potassium content in DAW601 of (elite x exotic) progeny on the first row, for α-amino nitrogen content in DAW601 of the (elite x exotic) progeny on the second row and for sodium content in DAW601 of the (elite x exotic) progeny on the third row. Note that the two steps can be the same. Stars in the step selected by eBIC represent SNPs detected and added into the model in previous steps

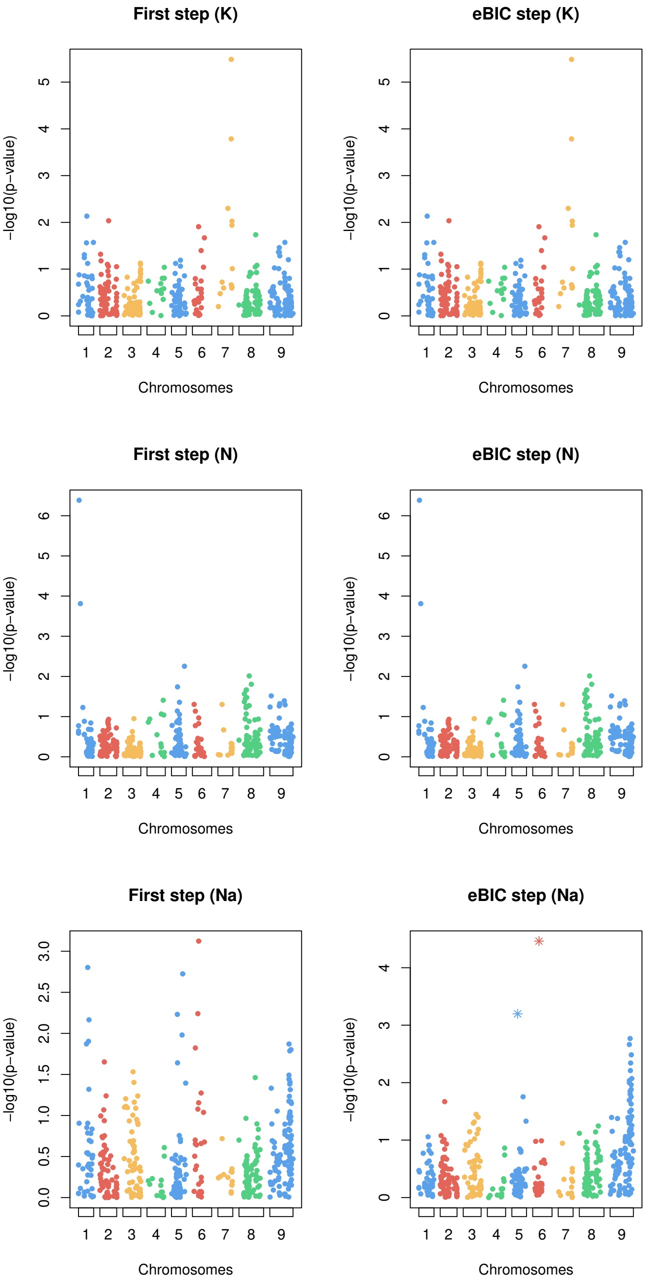

Fig. S13: Manhattan plots using the additive model of the first step of GWAS on the left, and the step selected by eBIC on the right for potassium content in DOM601 of (elite x exotic) progeny on the first row, for α-amino nitrogen content in DOM601 of the (elite x exotic) progeny on the second row and for sodium content in DOM601 of the (elite x exotic) progeny on the third row. Note that the two steps can be the same. Stars in the step selected by eBIC represent SNPs detected and added into the model in previous steps

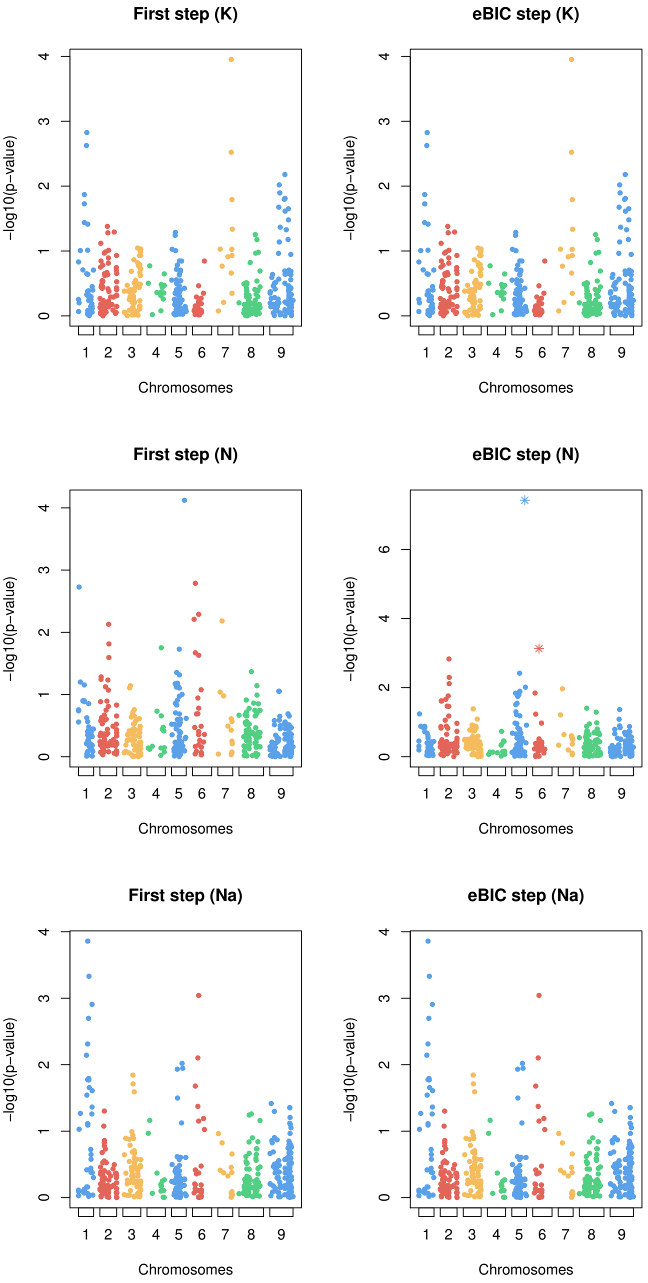

Fig. S14: Manhattan plots using the additive model of the first step of GWAS on the left, and the step selected by eBIC on the right for potassium content in PIE601 of (elite x exotic) progeny on the first row, for α-amino nitrogen content in PIE601 of the (elite x exotic) progeny on the second row and for sodium content in PIE601 of the (elite x exotic) progeny on the third row. Note that the two steps can be the same. Stars in the step selected by eBIC represent SNPs detected and added into the model in previous steps

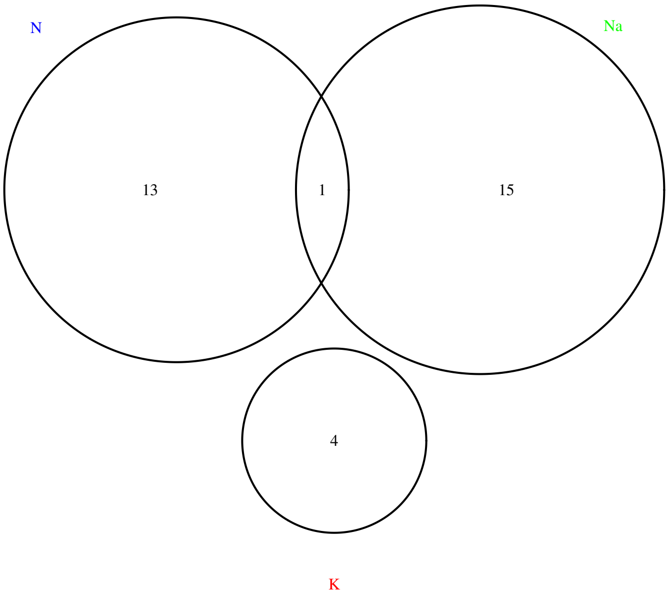

Fig. S15: Venn diagram of detected SNPs in the (elite x exotic) progeny for potassium content (K; meq/100g), sodium content (Na; meq/100g), and α-amino nitrogen content (N; meq/100g)

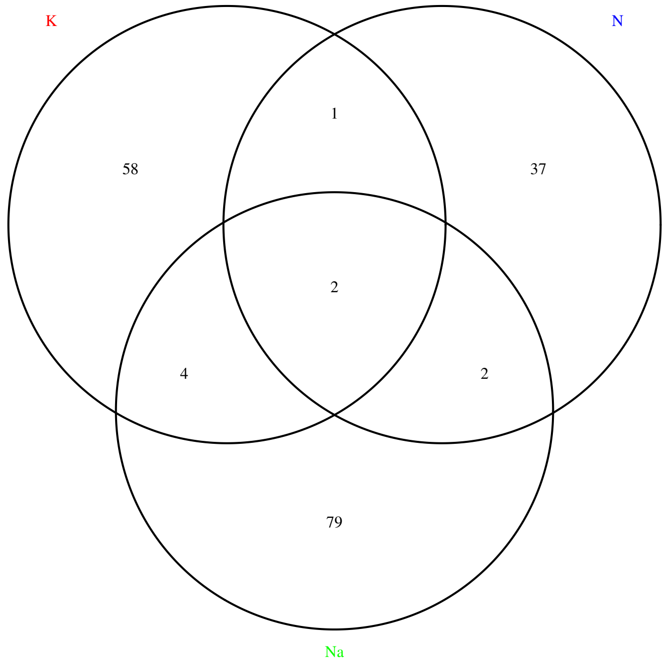

Fig. S16: Venn diagram of detected SNPs in the elite panel for potassium content (K; meq/100g), sodium content (Na; meq/100g), and α-amino nitrogen content (N; meq/100g)

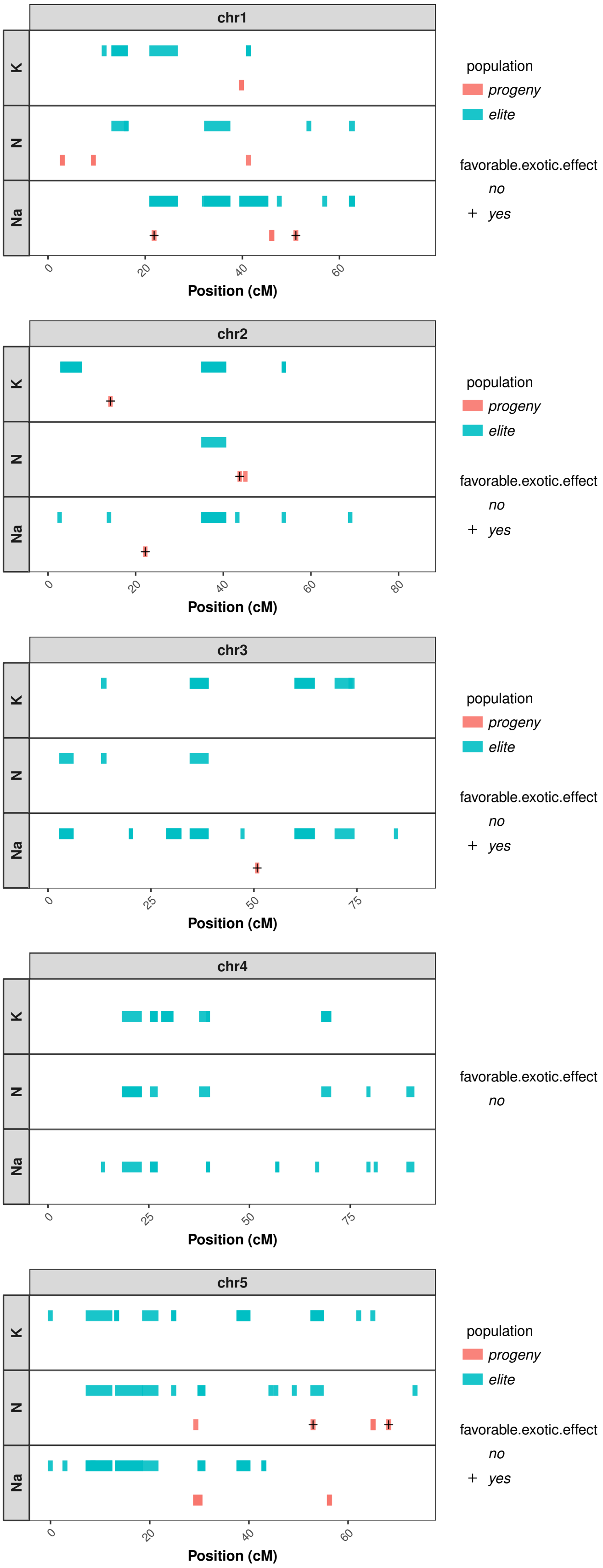

Fig. S17: QTL detected in (elite x exotic) progeny and in elite panel on each chromosome for potassium content (K; meq/100g), sodium content (Na; meq/100g), and α-amino nitrogen content (N; meq/100g). QTLs with favorable effect of the exotic allele were represented with the plus sign.

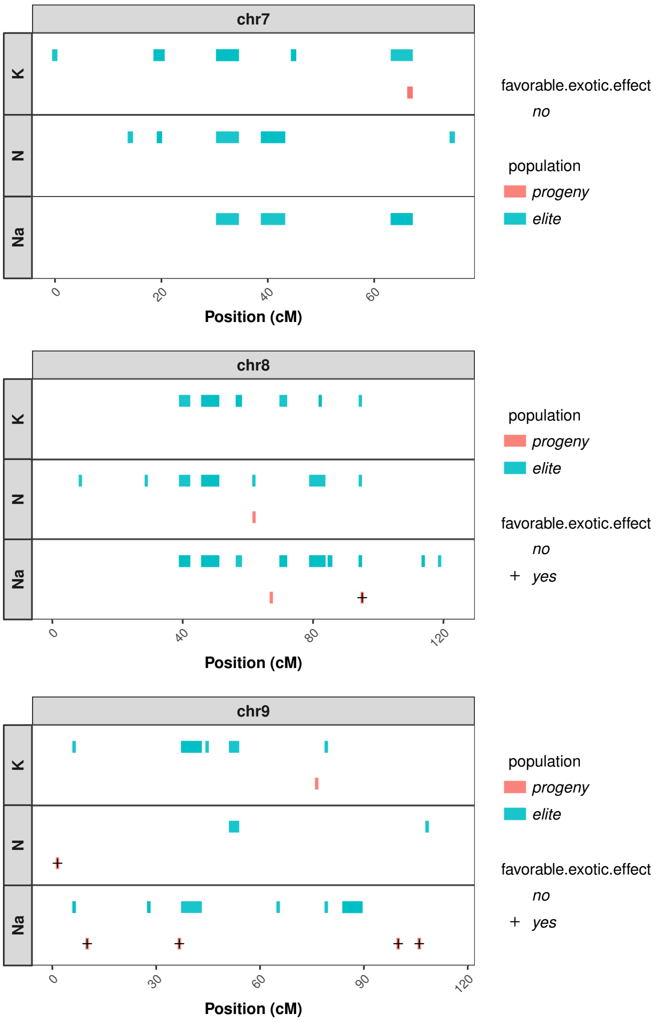

Fig. S17: QTL detected in (elite x exotic) progeny and in elite panel on each chromosome for potassium content (K; meq/100g), sodium content (Na; meq/100g), and α-amino nitrogen content (N; meq/100g). QTLs with favorable effect of the exotic allele were represented with the plus sign.

Table S1: SNPs associated with potassium content in the six environments and the mean phenotype of (elite x exotic) progeny. These SNPs are detected in association studies with an additive model (A) and an additive and dominance model (AD), selected with the eBIC criterion and merged into QTLs. Their position on chromosome, the proportion of variance they explained in the multi SNPs model selected by eBIC (%var), and information about the favorable or unfavorable effect of the exotic allele are also given

SNP	QTL	Environment	Model	Chr	Position	%var	Favorable exotic
SNP_00267	QTL_10	BEL601	A	1	39.81	0.11	no
SNP_00267	QTL_10	DAW601	A	1	39.81	0.17	no
SNP_01273	QTL_28	BER601	A	2	14.29	0.07	yes
SNP_07975	QTL_16	mean phenotype	A	7	66.69	0.18	no
SNP_07975	QTL_16	mean phenotype	AD	7	66.69	0.18	no
SNP_07975	QTL_16	AVE607	A	7	66.69	0.17	no
SNP_07975	QTL_16	AVE607	AD	7	66.69	0.17	no
SNP_07975	QTL_16	BEL601	A	7	66.69	0.11	no
SNP_07975	QTL_16	BEL601	AD	7	66.69	0.16	no
SNP_07975	QTL_16	BER601	A	7	66.69	0.16	no
SNP_07975	QTL_16	DOM601	A	7	66.69	0.14	no
SNP_07975	QTL_16	DOM601	AD	7	66.69	0.14	no
SNP_07975	QTL_16	PIE601	A	7	66.69	0.13	no
SNP_10155	QTL_22	BER601	A	9	76.34	0.12	no

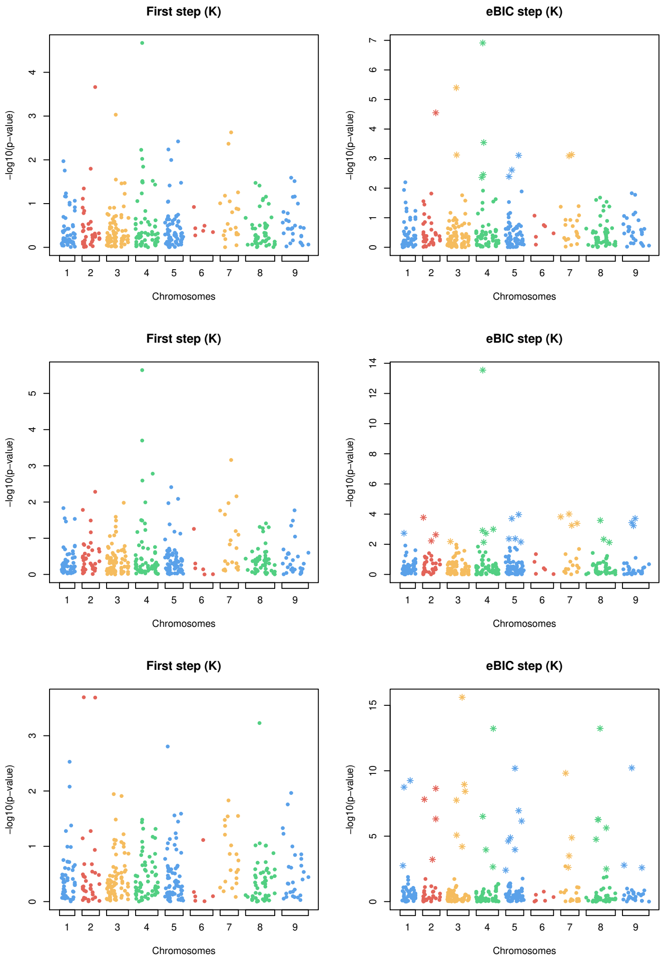

Fig. S18: Manhattan plots using the additive model of the first step of GWAS on the left, and the step selected by eBIC on the right for potassium content in the entire elite panel on the first row, for the panel A cluster on the second row, and for the panel B cluster on the third row. Note that the two steps can be the same. Stars in the step selected by eBIC represent SNPs detected and added into the model in previous steps

Table S2: SNPs associated with α-amino nitrogen content in the six environments and the mean phenotype of (elite x exotic) progeny. These SNPs are detected in association studies with an additive model (A) and an additive and dominance model (AD), selected with the eBIC criterion and merged into QTLs. Their position on chromosome, the proportion of variance they explained in the multi SNPs model selected by eBIC (%var), and information about the favorable or unfavorable effect of the exotic allele are also given

SNP	QTL	Environment	model	Chr	Position	%var	Favorable exotic
SNP_00018	QTL_26	BER601	A	1	2.99	0.16	no
SNP_00018	QTL_26	DOM601	A	1	2.99	0.23	no
SNP_00018	QTL_26	DOM601	AD	1	2.99	0.23	no
SNP_00041	QTL_27	AVE607	A	1	9.38	0.13	no
SNP_00041	QTL_27	DAW601	A	1	9.38	0.12	no
SNP_00274	QTL_12	DAW601	A	1	41.25	0.07	no
SNP_02037	QTL_05	PIE601	A	2	43.73	0.09	yes
SNP_02055	QTL_25	DAW601	A	2	45.03	0.03	no
SNP_05482	QTL_07	DAW601	A	5	29.31	0.04	no
SNP_06223	QTL_01	BEL601	A	5	52.91	0.10	yes
SNP_06319	QTL_01	mean phenotype	A	5	64.98	0.52	no
SNP_06319	QTL_01	BEL601	A	5	64.98	0.12	no
SNP_06319	QTL_01	DAW601	A	5	64.98	0.07	no
SNP_06319	QTL_01	PIE601	A	5	64.98	0.46	no
SNP_06344	QTL_29	mean phenotype	A	5	68.13	0.18	yes
SNP_06609	QTL_09	DAW601	A	6	30.98	0.05	yes
SNP_06641	QTL_02	PIE601	A	6	31.43	0.37	yes
SNP_09218	QTL_17	BEL601	A	8	61.88	0.10	no
SNP_09789	QTL_23	DAW601	A	9	1.57	0.10	yes
SNP_10930	QTL_30	BEL601	A	-	-	0.05	no

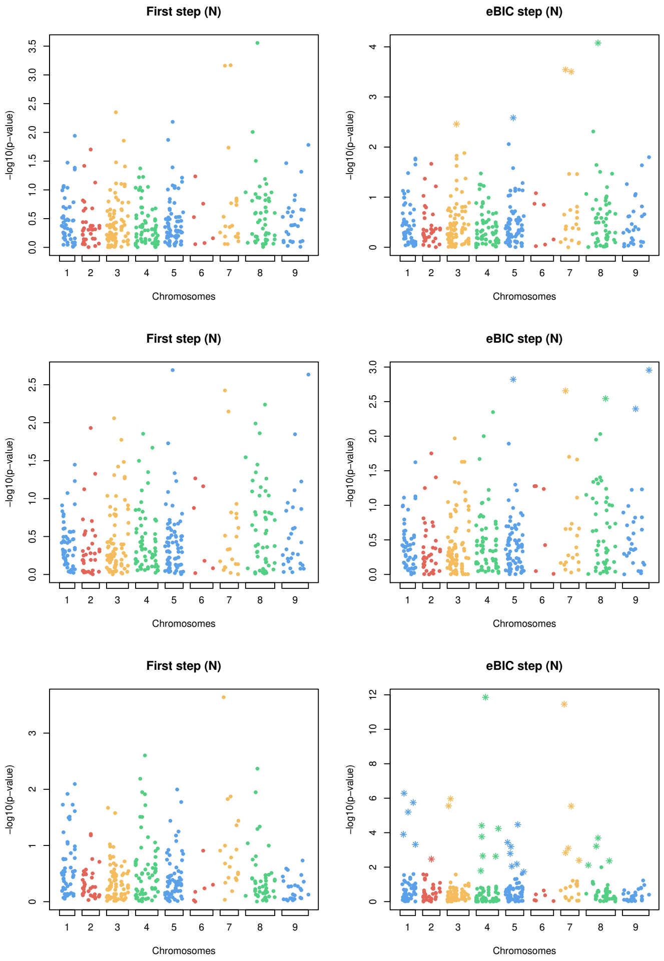

Fig. S19: Manhattan plots using the additive model of the first step of GWAS on the left, and the step selected by eBIC on the right for α-amino nitrogen content in the entire elite panel on the first row, for the panel A cluster on the second row, and for the panel B cluster on the third row. Note that the two steps can be the same. Stars in the step selected by eBIC represent SNPs detected and added into the model in previous steps

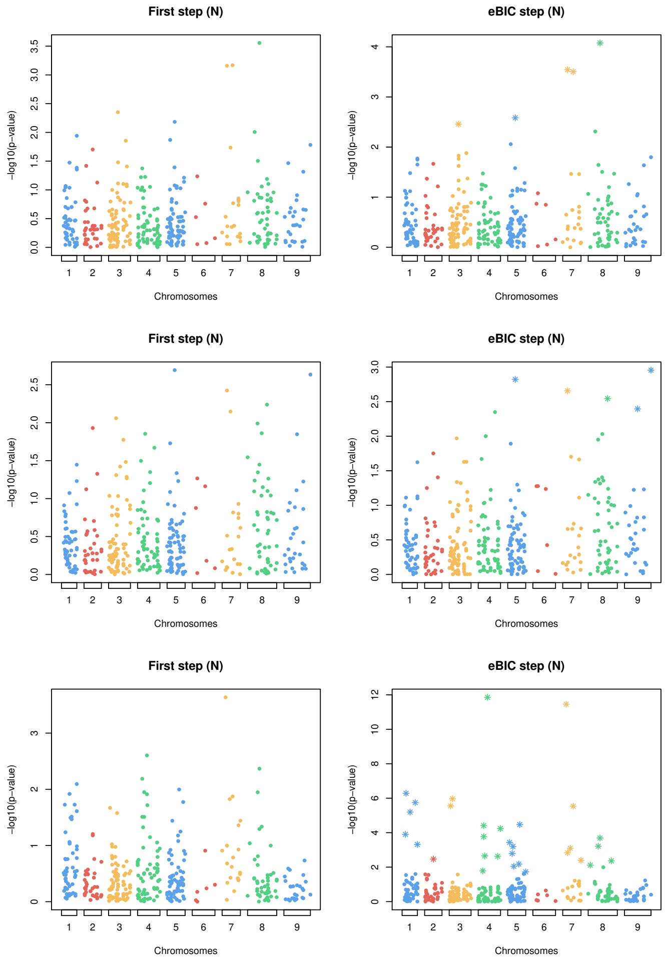

Fig. S20: Manhattan plots using the additive model of the first step of GWAS on the left, and the step selected by eBIC on the right for sodium content in the entire elite panel on the first row, for the panel A cluster on the second row, and for the panel B cluster on the third row. Note that the two steps can be the same. Stars in the step selected by eBIC represent SNPs detected and added into the model in previous steps

Table S3: SNPs associated with sodium content in the six environments and the mean phenotype of (elite x exotic) progeny. These SNPs are detected in association studies with an additive model (A) and an additive and dominance model (AD), selected with the eBIC criterion and merged into QTLs. Their position on chromosome, the proportion of variance they explained in the multi SNPs model selected by eBIC (%var), and information about the favorable or unfavorable effect of the exotic allele are also given

SNP	QTL	Environment	model	Chr	Position	%var	Favorable exotic
SNP_00116	QTL_14	mean phenotype	A	1	21.86	0.08	yes
SNP_00322	QTL_15	mean phenotype	A	1	46.05	0.20	no
SNP_00322	QTL_15	AVE607	A	1	46.05	0.17	no
SNP_00322	QTL_15	BER601	A	1	46.05	0.05	no
SNP_00322	QTL_15	PIE601	A	1	46.05	0.19	no
SNP_00350	QTL_06	mean phenotype	A	1	51.00	0.04	yes
SNP_01689	QTL_13	mean phenotype	A	2	22.23	0.02	yes
SNP_02804	QTL_04	mean phenotype	A	3	50.78	0.04	yes
SNP_05481	QTL_03	DOM601	A	5	29.26	0.07	no
SNP_05508	QTL_03	mean phenotype	A	5	30.14	0.07	no
SNP_06273	QTL_11	mean phenotype	A	5	56.20	0.05	no
SNP_06273	QTL_11	BEL601	A	5	56.20	0.14	no
SNP_06273	QTL_11	BER601	A	5	56.20	0.10	no
SNP_06641	QTL_02	mean phenotype	AD	6	31.43	0.22	no
SNP_06641	QTL_02	DAW601	A	6	31.43	0.14	no
SNP_06641	QTL_02	DOM601	A	6	31.43	0.15	no
SNP_09271	QTL_18	mean phenotype	A	8	67.17	0.04	no
SNP_09633	QTL_08	mean phenotype	A	8	95.03	0.06	yes
SNP_09818	QTL_20	mean phenotype	A	9	10.12	0.06	yes
SNP_09973	QTL_24	mean phenotype	A	9	36.71	0.05	yes
SNP_10511	QTL_21	DOM601	A	9	99.86	0.12	yes
SNP_10753	QTL_19	mean phenotype	A	9	105.93	0.11	yes
SNP_10931	QTL_31	mean phenotype	A	-	-	0.03	no

Table S4: SNPs associated with potassium content in the elite panel and in each on the two cluster (Panel A and Panel B). These SNPs are detected in association studies with an additive model (A) and an additive and dominance model (AD), selected with the eBIC criterion and merged into QTLs. Their position on chromosome and the proportion of variance they explained in the multi SNPs model selected by eBIC (%var) are also given

SNP	QTL	Panel	Model	Chr	Position	%var
SNP_00050	QTL_01	Panel A	A	1	11.58	0.01
SNP_00066	QTL_02	Panel A	A	1	15.96	0.03
SNP_00066	QTL_02	Panel B	A	1	15.96	0.02
SNP_00112	QTL_04	Panel	A	1	21.37	0.03
SNP_00273	QTL_08	Panel A	AD	1	41.25	0.03
SNP_00273	QTL_08	Panel A	A	1	41.25	0.03
SNP_01096	QTL_14	Panel B	A	2	3.36	0.02
SNP_01157	QTL_14	Panel A	AD	2	7.28	0.03
SNP_01157	QTL_14	Panel A	A	2	7.28	0.03
SNP_01951	QTL_16	Panel B	A	2	35.45	0.01
SNP_01993	QTL_16	Panel A	A	2	40.18	0.01
SNP_02116	QTL_18	Panel A	A	2	53.80	0.04
SNP_02117	QTL_19	Panel A	A	2	53.80	0.04
SNP_02117	QTL_19	Panel B	A	2	53.80	0.02
SNP_02117	QTL_19	Panel	A	2	53.80	0.04
SNP_02117	QTL_19	Panel	AD	2	53.80	0.04
SNP_02362	QTL_22	Panel B	A	3	13.40	0.01
SNP_02587	QTL_25	Panel B	A	3	37.14	0.01
SNP_02588	QTL_25	Panel	A	3	37.40	0.05
SNP_02591	QTL_25	Panel A	A	3	37.83	0.02
SNP_02602	QTL_25	Panel	A	3	38.51	0.03
SNP_02605	QTL_25	Panel A	A	3	38.51	0.03
SNP_03077	QTL_27	Panel A	A	3	61.05	0.54
SNP_03078	QTL_27	Panel A	A	3	61.05	0.07
SNP_03467	QTL_28	Panel A	A	3	70.06	0.15
SNP_03570	QTL_29	Panel A	A	3	73.40	0.04
SNP_04031	QTL_32	Panel	A	4	22.79	0.05
SNP_04038	QTL_33	Panel B	A	4	25.78	0.01
SNP_04049	QTL_33	Panel B	AD	4	26.75	0.01
SNP_04049	QTL_33	Panel B	A	4	26.75	0.13
SNP_04049	QTL_33	Panel	A	4	26.75	0.07
SNP_04049	QTL_33	Panel	AD	4	26.75	0.07
SNP_04050	QTL_33	Panel A	A	4	26.75	0.02
SNP_04052	QTL_34	Panel	A	4	28.64	0.03
SNP_04054	QTL_34	Panel	A	4	30.63	0.03
SNP_04054	QTL_34	Panel B	A	4	30.63	0.02
SNP_04086	QTL_36	Panel B	A	4	39.68	0.01
SNP_04087	QTL_35	Panel A	A	4	39.70	0.02
SNP_04815	QTL_39	Panel A	A	4	68.26	0.01
SNP_04881	QTL_39	Panel A	A	4	69.75	0.05
SNP_04881	QTL_39	Panel B	A	4	69.75	0.01
SNP_05218	QTL_43	Panel A	A	5	0.00	0.01
SNP_05276	QTL_45	Panel A	A	5	10.54	0.03
SNP_05308	QTL_47	Panel	A	5	13.35	0.01
SNP_05308	QTL_47	Panel B	A	5	13.35	0.01
SNP_05380	QTL_49	Panel A	A	5	19.19	0.02
SNP_05421	QTL_50	Panel B	A	5	24.86	0.01
SNP_05421	QTL_50	Panel	A	5	24.86	0.02
SNP_05554	QTL_52	Panel A	A	5	38.00	0.06
SNP_05555	QTL_52	Panel A	A	5	38.00	0.03
SNP_05559	QTL_52	Panel B	A	5	38.85	0.02
SNP_06220	QTL_57	Panel B	A	5	52.86	0.02
SNP_06220	QTL_57	Panel	A	5	52.86	0.03
SNP_06221	QTL_57	Panel A	A	5	52.86	0.02
SNP_06294	QTL_58	Panel B	A	5	62.09	0.01

SNP_06318	QTL_59	Panel A	A	5	64.94	0.02
SNP_07319	QTL_62	Panel B	A	7	0.00	0.02
SNP_07407	QTL_64	Panel A	A	7	19.03	0.01
SNP_07412	QTL_64	Panel A	A	7	20.13	0.03
SNP_07436	QTL_66	Panel A	A	7	30.77	0.01
SNP_07437	QTL_66	Panel	A	7	34.07	0.03
SNP_07437	QTL_66	Panel B	A	7	34.07	0.02
SNP_07437	QTL_66	Panel A	A	7	34.07	0.03
SNP_07445	QTL_68	Panel B	A	7	44.83	0.01
SNP_07445	QTL_68	Panel	A	7	44.83	0.03
SNP_07445	QTL_68	Panel A	A	7	44.83	0.01
SNP_07861	QTL_69	Panel B	A	7	66.69	0.01
SNP_08387	QTL_73	Panel A	A	8	40.52	0.05
SNP_08599	QTL_74	Panel A	A	8	48.34	0.03
SNP_08647	QTL_74	Panel A	A	8	49.07	0.03
SNP_09058	QTL_75	Panel A	A	8	56.77	0.11
SNP_09129	QTL_75	Panel B	A	8	57.69	0.03
SNP_09284	QTL_77	Panel B	A	8	71.54	0.01
SNP_09453	QTL_79	Panel A	A	8	82.16	0.01
SNP_09454	QTL_79	Panel A	A	8	82.16	0.05
SNP_09623	QTL_81	Panel B	A	8	94.45	0.01
SNP_09814	QTL_84	Panel A	A	9	6.35	0.01
SNP_09982	QTL_86	Panel A	A	9	37.70	0.05
SNP_09983	QTL_86	Panel B	A	9	37.70	0.03
SNP_10026	QTL_87	Panel B	A	9	44.72	0.03
SNP_10059	QTL_88	Panel B	A	9	51.51	0.02
SNP_10176	QTL_90	Panel A	A	9	79.10	0.01
SNP_10932	QTL_96	Panel B	A	-	-	0.08
SNP_10932	QTL_96	Panel B	AD	-	-	0.08

Table S5: SNPs associated with α-amino nitrogen content in the elite panel and in each on the two cluster (Panel A and Panel B). These SNPs are detected in association studies with an additive model (A) and an additive and dominance model (AD), selected with the eBIC criterion and merged into QTLs. Their position on chromosome and the proportion of variance they explained in the multi SNPs model selected by eBIC (%var) are also given

SNP	QTL	Panel	Model	Chr	Position	%var
SNP_00052	QTL_02	Panel A	A	1	13.56	0.02
SNP_00068	QTL_03	Panel A	A	1	16.14	0.07
SNP_00193	QTL_06	Panel A	A	1	32.63	0.06
SNP_00374	QTL_10	Panel A	A	1	53.68	0.04
SNP_00581	QTL_12	Panel A	A	1	62.43	0.03
SNP_01953	QTL_16	Panel A	A	2	35.88	0.01
SNP_02310	QTL_21	Panel A	A	3	5.77	0.04
SNP_02365	QTL_22	Panel A	A	3	13.74	0.04
SNP_02591	QTL_25	Panel	A	3	37.83	0.02
SNP_04004	QTL_32	Panel A	A	4	18.84	0.02
SNP_04027	QTL_32	Panel A	A	4	22.40	0.06
SNP_04030	QTL_32	Panel A	A	4	22.75	0.08
SNP_04049	QTL_33	Panel A	A	4	26.75	0.03
SNP_04076	QTL_35	Panel A	A	4	38.00	0.08
SNP_04816	QTL_39	Panel B	A	4	68.45	0.01
SNP_05085	QTL_40	Panel A	A	4	79.46	0.03
SNP_05203	QTL_42	Panel A	A	4	90.35	0.03
SNP_05272	QTL_45	Panel A	A	5	7.63	0.02
SNP_05358	QTL_48	Panel A	A	5	18.20	0.02
SNP_05404	QTL_49	Panel A	A	5	21.29	0.01
SNP_05421	QTL_50	Panel A	A	5	24.86	0.01
SNP_05510	QTL_51	Panel	A	5	30.73	0.02
SNP_05510	QTL_51	Panel B	A	5	30.73	0.03

SNP_06065	QTL_54	Panel A	AD	5	44.42	0.02
SNP_06080	QTL_55	Panel A	A	5	45.42	0.01
SNP_06185	QTL_56	Panel A	A	5	49.13	0.03
SNP_06254	QTL_57	Panel A	AD	5	54.56	0.02
SNP_06369	QTL_60	Panel A	A	5	73.44	0.01
SNP_07379	QTL_63	Panel A	A	7	14.19	0.05
SNP_07411	QTL_65	Panel B	A	7	19.65	0.02
SNP_07411	QTL_65	Panel A	A	7	19.65	0.01
SNP_07411	QTL_65	Panel	A	7	19.65	0.01
SNP_07436	QTL_66	Panel A	A	7	30.77	0.02
SNP_07440	QTL_67	Panel	A	7	42.76	0.03
SNP_07440	QTL_67	Panel A	A	7	42.76	0.04
SNP_08172	QTL_70	Panel A	A	7	74.61	0.01
SNP_08176	QTL_71	Panel A	A	8	8.66	0.01
SNP_08233	QTL_72	Panel	A	8	28.86	0.02
SNP_08402	QTL_73	Panel A	A	8	41.14	0.03
SNP_08599	QTL_74	Panel A	A	8	48.34	0.03
SNP_08599	QTL_74	Panel	A	8	48.34	0.03
SNP_09191	QTL_76	Panel A	A	8	61.83	0.02
SNP_09404	QTL_78	Panel B	A	8	79.27	0.03
SNP_09624	QTL_81	Panel A	A	8	94.45	0.05
SNP_10076	QTL_88	Panel B	A	9	53.48	0.04
SNP_10814	QTL_92	Panel B	A	9	108.19	0.02
SNP_10933	QTL_93	Panel A	A	-	-	0.05

Table S6: SNPs associated with sodium content in the elite panel and in each on the two cluster (Panel A and Panel B). These SNPs are detected in association studies with an additive model (A) and an additive and dominance model (AD), selected with the eBIC criterion and merged into QTLs. Their position on chromosome and the proportion of variance they explained in the multi SNPs model selected by eBIC (%var) are also given

SNP	QTL	Panel	Model	Chr	Position	%var
SNP_00119	QTL_04	Panel B	A	1	22.40	0.02
SNP_00159	QTL_04	Panel	A	1	26.22	0.06
SNP_00191	QTL_05	Panel B	A	1	32.19	0.01
SNP_00229	QTL_06	Panel A	A	1	33.00	0.08
SNP_00229	QTL_06	Panel A	AD	1	33.00	0.08
SNP_00229	QTL_06	Panel B	A	1	33.00	0.01
SNP_00260	QTL_06	Panel B	A	1	37.03	0.01
SNP_00268	QTL_07	Panel	A	1	39.87	0.08
SNP_00302	QTL_07	Panel A	A	1	44.82	0.03
SNP_00334	QTL_09	Panel	A	1	47.59	0.01
SNP_00426	QTL_11	Panel B	A	1	56.92	0.01
SNP_00581	QTL_12	Panel A	A	1	62.43	0.05
SNP_00581	QTL_12	Panel A	AD	1	62.43	0.05
SNP_00583	QTL_12	Panel	A	1	62.43	0.03
SNP_00583	QTL_12	Panel B	A	1	62.43	0.03
SNP_00657	QTL_12	Panel A	A	1	62.63	0.01
SNP_01088	QTL_13	Panel B	A	2	2.70	0.02
SNP_01255	QTL_15	Panel A	A	2	13.94	0.01
SNP_01953	QTL_16	Panel B	A	2	35.88	0.04
SNP_01953	QTL_16	Panel	A	2	35.88	0.02
SNP_02030	QTL_17	Panel A	A	2	43.19	0.01
SNP_02117	QTL_19	Panel B	A	2	53.80	0.01
SNP_02212	QTL_20	Panel B	A	2	68.92	0.01
SNP_02305	QTL_21	Panel B	A	3	3.27	0.03
SNP_02307	QTL_21	Panel B	A	3	3.27	0.08
SNP_02429	QTL_23	Panel B	A	3	20.15	0.01
SNP_02429	QTL_23	Panel	A	3	20.15	0.01
SNP_02489	QTL_24	Panel A	A	3	29.22	0.02
SNP_02497	QTL_24	Panel B	A	3	30.63	0.01

SNP_02510	QTL_24	Panel A	A	3	31.27	0.02
SNP_02510	QTL_24	Panel A	AD	3	31.27	0.02
SNP_02521	QTL_24	Panel A	A	3	31.89	0.03
SNP_02560	QTL_25	Panel A	A	3	34.89	0.10
SNP_02560	QTL_25	Panel A	AD	3	34.89	0.10
SNP_02743	QTL_26	Panel B	A	3	47.21	0.01
SNP_03040	QTL_27	Panel A	A	3	60.31	0.02
SNP_03078	QTL_27	Panel A	A	3	61.05	0.07
SNP_03088	QTL_27	Panel B	A	3	64.27	0.02
SNP_03623	QTL_28	Panel A	A	3	73.89	0.03
SNP_03822	QTL_30	Panel B	A	3	84.43	0.00
SNP_03988	QTL_31	Panel A	A	4	13.69	0.01
SNP_04027	QTL_32	Panel A	A	4	22.40	0.02
SNP_04049	QTL_33	Panel	A	4	26.75	0.02
SNP_04049	QTL_33	Panel B	A	4	26.75	0.01
SNP_04086	QTL_36	Panel B	A	4	39.68	0.01
SNP_04086	QTL_36	Panel	A	4	39.68	0.01
SNP_04302	QTL_37	Panel B	AD	4	56.88	0.01
SNP_04302	QTL_37	Panel B	A	4	56.88	0.01
SNP_04715	QTL_38	Panel A	A	4	66.74	0.02
SNP_05081	QTL_40	Panel A	A	4	79.46	0.01
SNP_05152	QTL_41	Panel B	A	4	81.27	0.01
SNP_05200	QTL_42	Panel B	A	4	89.37	0.02
SNP_05219	QTL_43	Panel A	A	5	0.00	0.02
SNP_05242	QTL_44	Panel B	A	5	2.95	0.01
SNP_05271	QTL_45	Panel B	A	5	7.63	0.01
SNP_05273	QTL_46	Panel B	A	5	8.59	0.01
SNP_05278	QTL_45	Panel B	A	5	11.24	0.01
SNP_05295	QTL_45	Panel B	A	5	11.99	0.02
SNP_05315	QTL_48	Panel A	A	5	13.54	0.01
SNP_05364	QTL_48	Panel A	A	5	18.20	0.02
SNP_05379	QTL_49	Panel B	A	5	18.98	0.01
SNP_05507	QTL_51	Panel B	A	5	30.14	0.02
SNP_05509	QTL_51	Panel A	A	5	30.62	0.01
SNP_05561	QTL_52	Panel A	A	5	38.85	0.01
SNP_05561	QTL_52	Panel B	A	5	38.85	0.02
SNP_05913	QTL_52	Panel B	A	5	39.79	0.01
SNP_06013	QTL_53	Panel B	A	5	43.01	0.01
SNP_06014	QTL_53	Panel B	A	5	43.01	0.01
SNP_07235	QTL_61	Panel A	A	6	58.00	0.01
SNP_07436	QTL_66	Panel A	A	7	30.77	0.01
SNP_07439	QTL_67	Panel A	A	7	39.18	0.02
SNP_07813	QTL_69	Panel B	A	7	63.56	0.04
SNP_07813	QTL_69	Panel A	A	7	63.56	0.03
SNP_07813	QTL_69	Panel	A	7	63.56	0.03
SNP_07813	QTL_69	Panel	AD	7	63.56	0.03
SNP_08358	QTL_73	Panel B	A	8	39.38	0.03
SNP_08403	QTL_73	Panel A	A	8	41.14	0.03
SNP_08427	QTL_73	Panel A	AD	8	41.84	0.03
SNP_08428	QTL_73	Panel A	AD	8	41.84	0.02
SNP_08538	QTL_74	Panel B	A	8	46.19	0.02
SNP_08647	QTL_74	Panel	A	8	49.07	0.03
SNP_08647	QTL_74	Panel B	A	8	49.07	0.01
SNP_08712	QTL_74	Panel	A	8	50.72	0.04
SNP_09120	QTL_75	Panel B	A	8	57.60	0.01
SNP_09282	QTL_77	Panel A	AD	8	70.16	0.01
SNP_09282	QTL_77	Panel	A	8	70.16	0.01
SNP_09424	QTL_78	Panel B	A	8	80.73	0.04
SNP_09424	QTL_78	Panel	A	8	80.73	0.03
SNP_09481	QTL_78	Panel A	AD	8	83.26	0.03
SNP_09505	QTL_80	Panel A	A	8	84.95	0.07

SNP_09508	QTL_80	Panel A	A	8	85.35	0.11
SNP_09623	QTL_81	Panel A	A	8	94.45	0.05
SNP_09623	QTL_81	Panel	A	8	94.45	0.01
SNP_09623	QTL_81	Panel B	A	8	94.45	0.02
SNP_09744	QTL_82	Panel B	A	8	113.72	0.01
SNP_09744	QTL_82	Panel	A	8	113.72	0.01
SNP_09758	QTL_83	Panel A	A	8	118.74	0.02
SNP_09814	QTL_84	Panel A	AD	9	6.35	0.04
SNP_09814	QTL_84	Panel A	A	9	6.35	0.04
SNP_09929	QTL_85	Panel B	A	9	27.90	0.04
SNP_09929	QTL_85	Panel	A	9	27.90	0.04
SNP_09929	QTL_85	Panel	AD	9	27.90	0.04
SNP_10021	QTL_86	Panel B	A	9	42.70	0.01
SNP_10084	QTL_89	Panel B	A	9	65.22	0.01
SNP_10177	QTL_90	Panel B	A	9	79.10	0.01
SNP_10219	QTL_91	Panel A	A	9	84.27	0.01
SNP_10226	QTL_91	Panel A	AD	9	89.05	0.01
SNP_10226	QTL_91	Panel B	A	9	89.05	0.02
SNP_10226	QTL_91	Panel	A	9	89.05	0.04
SNP_10226	QTL_91	Panel	AD	9	89.05	0.04
SNP_10226	QTL_91	Panel A	A	9	89.05	0.08
SNP_10933	QTL_93	Panel A	A	-	-	0.04
SNP_10934	QTL_94	Panel	A	-	-	0.03
SNP_10934	QTL_94	Panel B	A	-	-	0.04
SNP_10935	QTL_95	Panel B	A	-	-	0.01
SNP_10935	QTL_95	Panel A	A	-	-	0.01
